# *Vibrio*-Sequins - dPCR-traceable DNA standards for quantitative genomics of *Vibrio spp*

**DOI:** 10.1186/s12864-023-09429-8

**Published:** 2023-07-04

**Authors:** Sabrina Flütsch, Fabian Wiestner, Lisa Butticaz, Dominik Moor, Kai N. Stölting

**Affiliations:** 1 Swiss Federal Institute of Metrology METAS, Lindenweg 50, Bern-Wabern, 3003 Switzerland; 2 Federal Food Safety and Veterinary Office FSVO, Schwarzenburgstrasse 165, Bern-Köniz, 3003 Switzerland; 3 Swiss Federal Institute of Metrology METAS, Campus Liebefeld, Schwarzenburgstrasse 165, Bern-Köniz, 3097 Switzerland

**Keywords:** *Vibrio* species, Quantitative genomics, NGS, DNA standards, Metrological traceability, dPCR, Measurement uncertainty

## Abstract

**Background:**

*Vibrio spp.* are a diverse group of ecologically important marine bacteria responsible for several foodborne outbreaks of gastroenteritis around the world. Their detection and characterization are moving away from conventional culture-based methods towards next generation sequencing (NGS)-based approaches. However, genomic methods are relative in nature and suffer from technical biases arising from library preparation and sequencing. Here, we introduce a quantitative NGS-based method that enables the quantitation of *Vibrio spp.* at the limit of quantification (LOQ) through artificial DNA standards and their absolute quantification *via* digital PCR (dPCR).

**Results:**

We developed six DNA standards, called *Vibrio-*Sequins, together with optimized TaqMan assays for their quantification in individually sequenced DNA libraries *via* dPCR. To enable *Vibrio*-Sequin quantification, we validated three duplex dPCR methods to quantify the six targets. LOQs were ranging from 20 to 120 cp/µl for the six standards, whereas the limit of detection (LOD) was ~ 10 cp/µl for all six assays. Subsequently, a quantitative genomics approach was applied to quantify *Vibrio*-DNA in a pooled DNA mixture derived from several *Vibrio* species in a proof-of-concept study, demonstrating the increased power of our quantitative genomic pipeline through the coupling of NGS and dPCR.

**Conclusions:**

We significantly advance existing quantitative (meta)genomic methods by ensuring metrological traceability of NGS-based DNA quantification. Our method represents a useful tool for future metagenomic studies aiming at quantifying microbial DNA in an absolute manner. The inclusion of dPCR into sequencing-based methods supports the development of statistical approaches for the estimation of measurement uncertainties (MU) for NGS, which is still in its infancy.

**Supplementary Information:**

The online version contains supplementary material available at 10.1186/s12864-023-09429-8.

## Background

*Vibrio*, a genus of ubiquitous, Gram-negative bacteria present in a wide range of temperate aquatic and marine habitats is an emerging concern for human health as water temperatures rise [1–5]. The genus consists of more than 100 described *Vibrio spp*., of which 12 are associated with human infections [3, 6]. The most common pathogenic species are *Vibrio cholerae* (*V. cholerae*), which can cause the severe diarrhoeal disease cholera, and the two non-cholera species *Vibrio parahaemolyticus* (*V. parahaemolyticus*) and *Vibrio vulnificus* (*V. vulnificus*), which are linked to vibriosis, e.g., wound infections, septicaemia and gastroenteritis [7]. Pathogen transmission to humans occurs predominantly through contact with polluted water and the consumption of contaminated seafood, mainly oysters [3]. Cases of documented *Vibrio spp.* infections follow seasonal distributions with most cases appearing during the warmer periods of the year [3, 5]. Exposure to these emerging foodborne pathogens is detrimental to public health [8] and therefore continuous and regular monitoring of seafood is essential to prevent *Vibrio* infection outbreaks. Generally, the detection of *Vibrio spp*. occurs through conventional culture-based methods. Such methods, however, are limited in terms of quantitative power, delivering solely information about presence or absence of *Vibrio spp*. Moreover, gold standard methods, such as the ISO 21872-1:2017 [9] are based on two rounds of culturing followed by biochemical confirmation and are therefore labour-intensive, time-consuming and introducing bias through culturing. Hence, a fast and unbiased method that can directly detect and quantify the pathogens from food samples without additional steps of culturing/PCR is desirable. Metagenomic next generation sequencing (NGS) can reveal the composition of microbial communities within samples without any *a priori* knowledge [10, 11]. NGS-based methods enable the detection of *Vibrio spp*. together with the determination of *Vibrio* strains as well as the presence of epidemiologically interesting genes, such as virulence and antibiotic resistance genes. However, despite huge efforts in advancing quantitative genomic approaches, including metagenomics, the analysis of such data remains challenging. Firstly, NGS suffers from technical biases arising through library preparation and sequencing [10, 12]. Secondly, metagenomic sequencing is relative in nature and several studies have highlighted the inherent limitations of relative abundance analyses (e.g., of microbial taxa) among samples [13–15]. To overcome such biases, great efforts have been made in developing methods for the absolute quantification of microbial taxa in environmental samples, which simultaneously control for technical biases. One such promising method is based on the spiking of artificial DNA standards of known concentrations to the samples before library preparation, allowing the quantification of microbial taxa through calibration and normalization approaches [16–18]. Internal reference standards permit the evaluation of technical variables that bias NGS-based analyses [18]. The method, however, suffers from its own limitations including amplification and sequencing biases affecting the standards. Importantly, quantitative (meta)genomic methods currently in use are not metrologically traceable, as these methods rely on standard quantification *via* fluorometric methods [12, 15, 18–23], which lack traceable reference methods and reference materials. Furthermore, if DNA standards are quantified using exclusively fluorometric methods prior to spiking them to the samples (e.g., before samples and standards undergo a combined library preparation), relevant changes in DNA standard concentrations in the final DNA libraries might be missed. However, these DNA standard concentrations are critical for the quantification of the concomitant microbial DNAs.

Here, we developed a NGS-based detection and quantification method for *Vibrio spp*. from environmental samples (e.g., seafood) allowing the quantification of *Vibrio*-DNA within samples at the limit of quantification (LOQ). To assure metrological traceability of our quantitative genomic approach, we extended existing methods of spiking DNA standards by applying the SI-traceable reference method digital PCR (dPCR) [24, 25] to quantify copy number concentrations (cp/µl) of the DNA standards in an absolute manner. We link the dPCR-based quantification of spiked standards in individual DNA libraries to the mathematical model for quantitative metagenomics developed by Li *et al*., 2021 [23]. For feasibility reasons, we developed only six artificial DNA standards – called *Vibrio-*Sequins - and each standard comes with its own optimised TaqMan assay and extensive dPCR method validation studies were performed for the quantification of the *Vibrio-*Sequins.

## Results

### Design and generation of ***Vibrio-***Sequins

We developed metrologically traceable DNA standards through absolute quantification *via* dPCR for the NGS-based detection and quantification of *Vibrio spp*. (predominantly *V. cholerae*, *V. parahaemolyticus* and *V. vulnificus*) in food samples (e.g., seafood). We designed and characterized a set of six artificial DNA sequences, called *Vibrio-*Sequins, following previously described concepts [18, 20, 22]. To optimally represent the features and characteristics of naturally occurring *Vibrio*-DNA sequences, *Vibrio-*Sequins were selected from *V. cholerae* core gene sequences present also in the genomes of *V. parahaemolyticus* and *V. vulnificus*, exhibiting high sequence similarity amongst the homologous genes. Four of the *Vibrio-*Sequin sequences are derived from actual *V. parahaemolyticus* (RIMD 2210633; GCA_000196095.1 ASM19609v1) gene sequences – *rplA, ushA, valS* and *xni* – representing the typical range of GC-contents of *Vibrio* genomes (44–47%). To broaden the range of covered GC-contents, two additional sequences were selected from the *V. parahaemolyticus* genome to span a lower (LC1; 27%) and a higher (HC1; 58%) GC-content than the average *Vibrio* genome. *Vibrio-*Sequins cover a range of different lengths (540–1156 bp), allowing the combined investigation of the effects of GC-contents on DNA library preparation and sequencing together with the impacts of sequence length (see additional file 2 for details on *Vibrio-*Sequin sequences). After selection of appropriate DNA fragments, the sequences were inverted (e.g., 3’ to 5’) to eliminate sequence similarity to naturally occurring sequences, while maintaining nucleotide composition, GC-content, length and DNA motifs [12, 18–22]. Subsequently, the inverted sequences were queried against the BLASTN non-redundant nt database to guarantee uniqueness of the *Vibrio-*Sequins. None of the six sequences provided significant similarity to sequences present in the nt database as of 13.09.2022. The inverted sequences were finalised by adding flanking sequences for T7 (forward) and T3 (reverse) primer-based full-length amplification (additional file 8 and additional file 3). *Vibrio-*Sequins were synthesised individually and delivered within linearized pUC57 plasmids, from which they could be full-length amplified (additional file 8 and additional file 3). The respective PCR-amplicons were purified and verified through Sanger sequencing prior to downstream analyses.

### ***Vibrio-***Sequin TaqMan assay design and optimization

dPCR is considered a SI-traceable reference measurement procedure [24, 25]. It is utilised for the counting of individual DNA molecules that have been partitioned into thousands of single reactions without employing reference materials [24, 26–29]. In this study, we applied dPCR to ensure metrological traceability of the absolute copy number concentration (cp/µl) quantifications of the *Vibrio-*Sequins within individual DNA libraries after enzymatic library preparation. These measurements were ultimately required for the quantification of the accompanying *Vibrio*-DNA in the libraries. In order to quantify the *Vibrio-*Sequins individually, we first developed a suitable hydrolysis probe-based (TaqMan) assay [30] for each of the standards. Henceforth, the term “assay” refers to a specific combination of forward and reverse PCR primers and a single combined quencher-fluorophore PCR probe. First, primer specificity was demonstrated in a conventional PCR. Therefore, *Vibrio*-Sequin standards (as dilutions of the original synthesised pUC57 plasmids) were spiked to a mixture of DNA extracts from cultured *Vibrio spp*. to reassemble “target” sequencing samples consisting of *Vibrio*-DNA extracts. Additional Fig. S1 shows a single PCR product for each of the six targets with the desired length, while no bands were observed in the no template controls (NTCs, containing PCR mix and H_2_O). Further information on amplicon size and location is provided in additional file 8. Primers and probes are listed in additional file 3.

Previous reports have demonstrated the impact of and the need for TaqMan assay optimisation on both LOD and LOQ [31–33]. We therefore optimised the individual TaqMan assays by means of quantitative PCR (qPCR) in terms of primer concentrations prior to using the assays in dPCR reactions. 13 different combinations of concentrations of forward and reverse primers for each of the six different assays were tested at otherwise identical conditions with a set probe concentration at 150 nM, identical cycling conditions and PCR master mix. We observed very different primer concentration optima for the individual six TaqMan assays (additional Fig. S2A-F). Optima (black traces; additional Fig. S2A-F) were characterised by low variation among the three technical replicates (SD), low C_T_ values and maximal normalized fluorescence amplitudes. Downstream analyses including PCR efficiency tests (additional file 3) and dPCR-based quantifications were performed with the optimal primer concentrations for each assay (additional Fig. S2A-F).

### dPCR method validations for the quantification of ***Vibrio-***Sequins

**Fig. 1:**
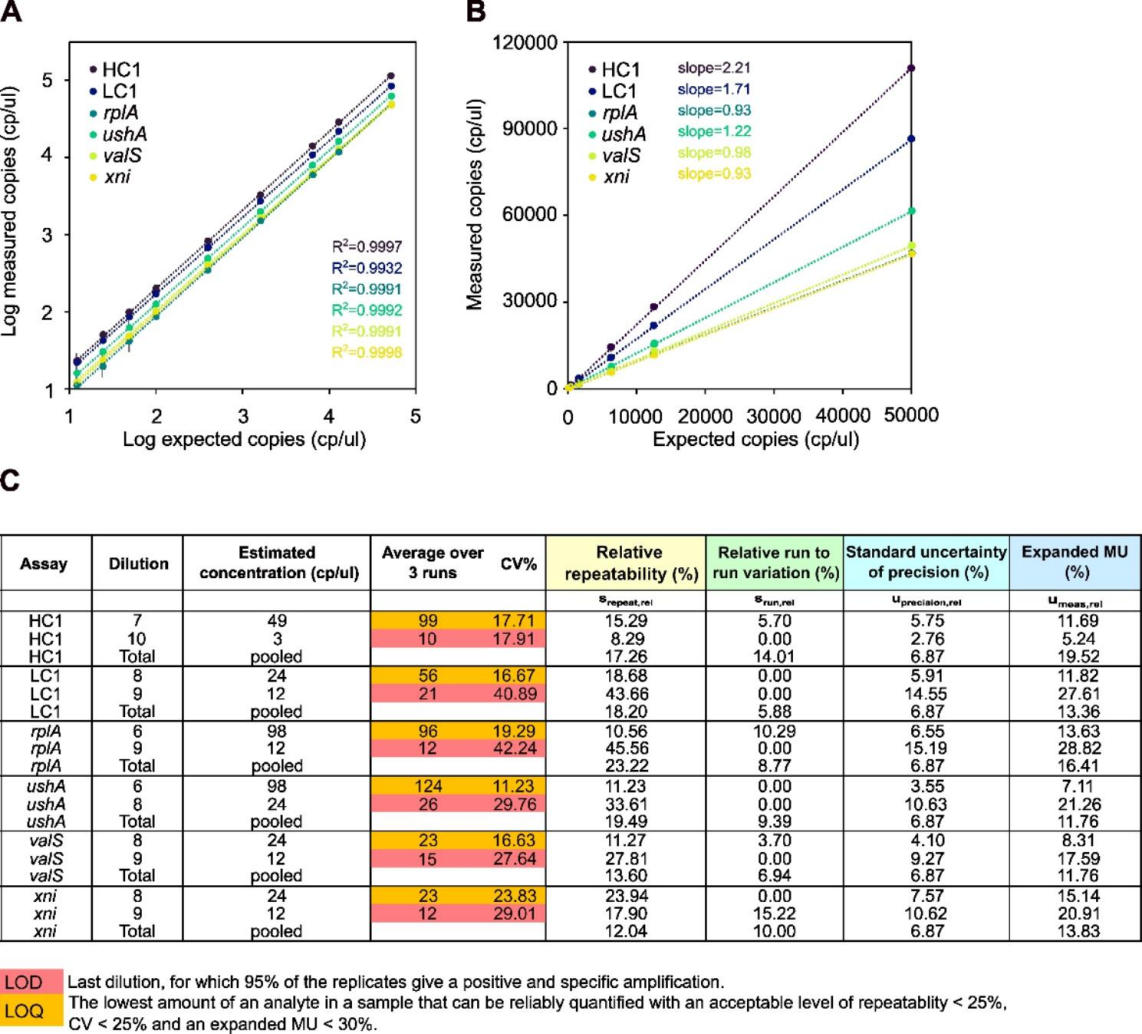
dPCR method validations. **(A)** Log-transformed linearity of the three duplex dPCR methods (HC1-LC1, *rplA*-*xni*, *ushA*-*valS*) for the quantification of *Vibrio-*Sequins within a *Vibrio*-DNA matrix. (**(B)** Untransformed linearity of the three dPCR methods (HC1-LC1, *rplA*-*xni*, *ushA*-*valS*) for the quantification of *Vibrio-*Sequins within a *Vibrio*-DNA matrix. Slopes indicate the discrepancies between Qubit and dPCR measurements for a given standard. (**A-B**) PCR-amplicons of the respective standards were measured for a copy number range of 3–50000 cp/µl. Data represent means ± expanded measurement uncertainty (MU) (n = 10; n_runs_ = 3). **(C)** Relevant results of the dPCR method validations for each *Vibrio-*Sequin standard. Data shown are the overall average dPCR-determined copy number concentrations (cp/µl) and the overall %CV over three runs, calculated relative repeatability (%), relative run-to-run variation (%), relative standard uncertainty related to precision (%) and the MU for *k* = 2 for the concentration levels determined as LODs and LOQs. In cases were MS_betweenrun_ < MS_withinrun_, the relative run-to-run variation was considered negligible compared to the relative repeatability and set to 0 [36]. Full table with results of the validation study is given in additional Fig. S4

To determine absolute copy number concentrations (cp/µl) of the individual *Vibrio-*Sequins using the optimised TaqMan assays, we developed and validated three duplex dPCR methods for the Stilla Naica crystal dPCR system (Stilla Technologies, France). The approach of method validation followed the ISO standard 20395 [34]. Primer specificity was tested by conventional PCR (see above; additional Fig. S1). The validation of the three dPCR methods for the quantification of *Vibrio-*Sequins in duplex mode – HC1 (Cy5) and LC1 (FAM), *rplA* (FAM) and xni (Cy5), *ushA* (Cy5.5) and *valS* (Cy3) - was carried out by measuring 10 different copy number concentrations (cp/µl) of each standard in a matrix of *Vibrio*-DNA (Fig. 1A-B). The matrix of *Vibrio*-DNA consisted of pooled DNA extracts from several cultured *Vibrio spp.* At each concentration level, 10 replicate measurements were performed. Replicates were distributed over three individual runs. Replicate measurements within a single run were performed under repeatability conditions – same analyst, same PCR mix, same batch of chips. All three assays delivered near perfect linearity as indicated by the R^2^ values for the individual standards (Fig. 1A), enabling the simultaneous measurement of six targets with three duplex reactions with the same confidence level. Additionally, the amplitude heat plots of the duplex dPCR reactions show clear separation among negative and positive partitions of droplets with little to almost no rain (additional Fig. S3), whereby rain describes the intermediate endpoint fluorescence values located between positive and negative clusters [35]. Figure 1B depicts the absolute copy numbers of the standards measured by the three dPCR methods against the expected values based on Qubit quantifications of the stock solutions of the standards. While the slopes for the standards *rplA, valS* and *xni* were close to 1, indicating a near perfect linear relationship between the two methods of quantification, there were obvious discrepancies for the measurements of the HC1, LC1 and *ushA* standards. The dPCR-measured copy numbers were 22%, 71% and 121% higher for *ushA*, LC1 and HC1 respectively (Fig. 1B).

The dPCR results for each *Vibrio-*Sequin were grouped per run and analysed by one-way analysis of variance (ANOVA) [34, 36, 37]. For each of the concentration levels, the relative repeatability (%), run-to-run variation (%), the relative uncertainty of precision (%) as well as the expanded measurement uncertainty (MU %, applying *k* = 2) were estimated according to the ISO standard 20395 [34]. Calculated values for all four parameters for each standard and each concentration level are shown in Fig. 1C and additional Fig. S4 along with the averages and coefficients of variation (CV%) for the individual runs and the overall averages and CV% summarizing all runs. Values obtained for each parameter were pooled into one summarizing value as described in [36]. The pooled relative repeatability remained below 20% for all targets, except for *rplA*, for which the relative repeatability was slightly higher (23.22%; Fig. 1C). The pooled run-to-run variation was estimated to be below or equal to 10%, except for the HC1 assay (14.01%; Fig. 1C). The pooled MU was below 20% for all six assays, whereby overall the assays for *valS* and *xni* were associated with the lowest MU. LOD and LOQ were also determined [34]. As depicted in Fig. 1C and additional Fig. S4, as LOD, we considered the lowest analyte concentration that can be distinguished from zero with 95% confidence. Hence, th LOD was determined as the concentration level at which at least three positive droplets were present in all 10 replicates. LOD values are marked in red in Fig. 1C. For all assays, the LODs were found to be below 10–25 cp/µl. The LOQ on the other hand was defined as the lowest concentration of the analyte that can be determined with an acceptable performance. We therefore defined the performance criteria as shown in Fig. 1C as relative repeatability < 25%, CV < 25% and MU < 30%. LOQs, marked in orange in Fig. 1C, were ~ 20 cp/µl for the *valS* and *xni* assays, whereas higher values of ~ 50 cp/µl were obtained for the HC1 and LC1 assays. Finally, for the *rplA* and *ushA* assays, the LOQs were ~ 100 and 120 cp/µl, respectively.

### Experimental validation of ***Vibrio-***Sequins

**Fig. 2:**
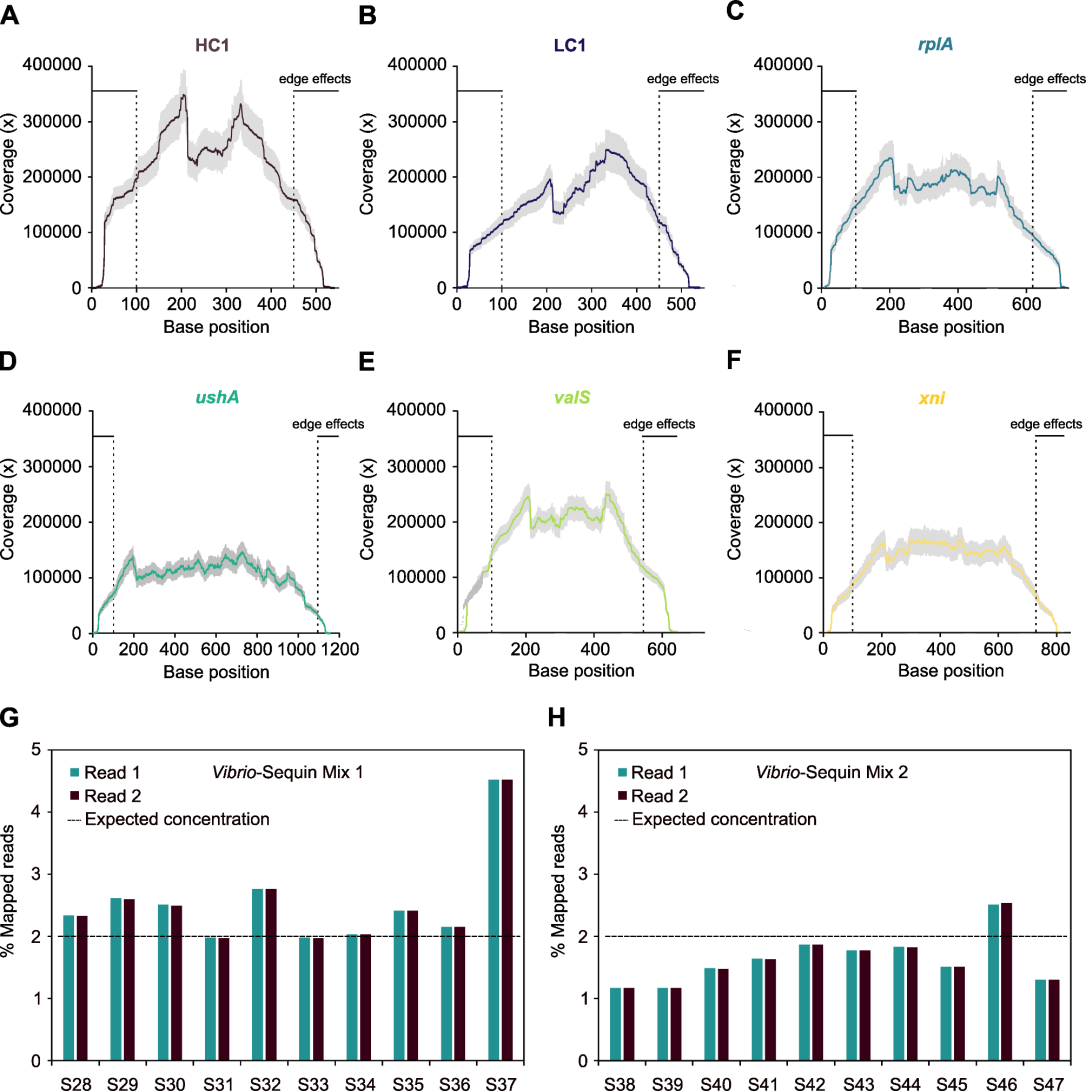
Technical variability between and within *Vibrio-*Sequin mixtures. **A-F)** Data show the average per-base coverages ± SD of the individual *Vibrio-*Sequins. Standards were sequenced pure and undiluted in triplicates (n = 3; S1-S3 *rplA, valS* and *xni*; S25-S27 HC1, LC1 and *ushA*). Terminal sequences of each *Vibrio-*Sequin (~ 100 bp) show impacts through edge effects and were excluded for downstream analyses. **G-H)** Histograms of the percentages of mapped reads (%) of the two *Vibrio-*Sequin mixes in each of the 10 spiked samples (*Vibrio-*Sequin mix 1 – S28-S37; *Vibrio-*Sequin mix 2 – S38-S47) to the *Vibrio-*Sequin reference sequences. Shown is the percentage of mapped reads for the individual reads (Read 1 and Read 2) within a library. The dashed line indicates the expected percentage of mapped reads (2%) to the *Vibrio-*Sequin reference sequences in each library

To investigate the experimental performance of the six *Vibrio-*Sequins, we initially sequenced a 1:1:1 mixture (40 ng DNA each; additional file 4) of the *rplA, valS* and *xni* standards (samples S1-S3) and of the HC1, LC1 and *ushA* standards (samples S25-S28) without the addition of any natural DNA. The sequencing of the pure, undiluted standard mixtures was performed in triplicates to examine the quantitative accuracy and magnitude of variability among the replicates caused by the processes of library preparation, sequencing and alignment. The resulting sequencing reads were aligned against a combined index containing the *Vibrio-*Sequin sequences along with the reference *Vibrio* genome sequences and the average per-base coverage of the individual standards was visualised (Fig. 2A-F). All six standard sequences were highly covered and the variation among the replicates was small, resulting in comparable coverage profiles (Fig. 2A-F and additional Fig. S5). Edge effects – reduction of sequencing coverage - at each border of the standards were observed as described previously [12, 18, 21]. We therefore, removed 100 bp from each end of the boarders of the standards for downstream analyses [12, 18, 21]. Since the sequencing of the pure, undiluted standards revealed that the *Vibrio-*Sequin-derived reads were affected consistently, we sought to further investigate the impact of library preparation and sequencing on “realistic” *Vibrio*-DNA samples (samples S28-S47; additional file 4). We therefore generated *Vibrio-*Sequin mixes 1 and 2 (for details see additional file 6), each containing all six standards in distinct concentrations to span a total concentration range of ~ 3.3 × 10^5^-fold, permitting the determination of the LOD of *Vibrio-*Sequin sequencing. Prior to library preparation, *Vibrio-*Sequin mix 1 was spiked to samples S28-S37 at a 2% fractional abundance, while samples S38-S47 were spiked with 2% of *Vibrio-*Sequin mix 2. The combined sample/*Vibrio-*Sequin mixture then underwent library preparation and sequencing. Subsequently, the obtained sequencing reads were mapped against the earlier mentioned combined index. As shown in Fig. 2G and H, sample composition and quality strongly affected library preparation and sequencing, as we obtained different percentages of mapped reads to *Vibrio-*Sequin reference sequences for individual libraries, although all libraries contained the same amount of standards (2%). The dashed lines in Fig. 2G H indicate the expected 2% of reads mapping to *Vibrio-*Sequin sequences. However, most libraries contained slightly more or less than the expected percentage of the *Vibrio-*Sequin reads, suggesting that the differences are derived from unequal processing during library preparation and/or sequencing (cluster generation) for the individual samples.

**Fig. 3:**
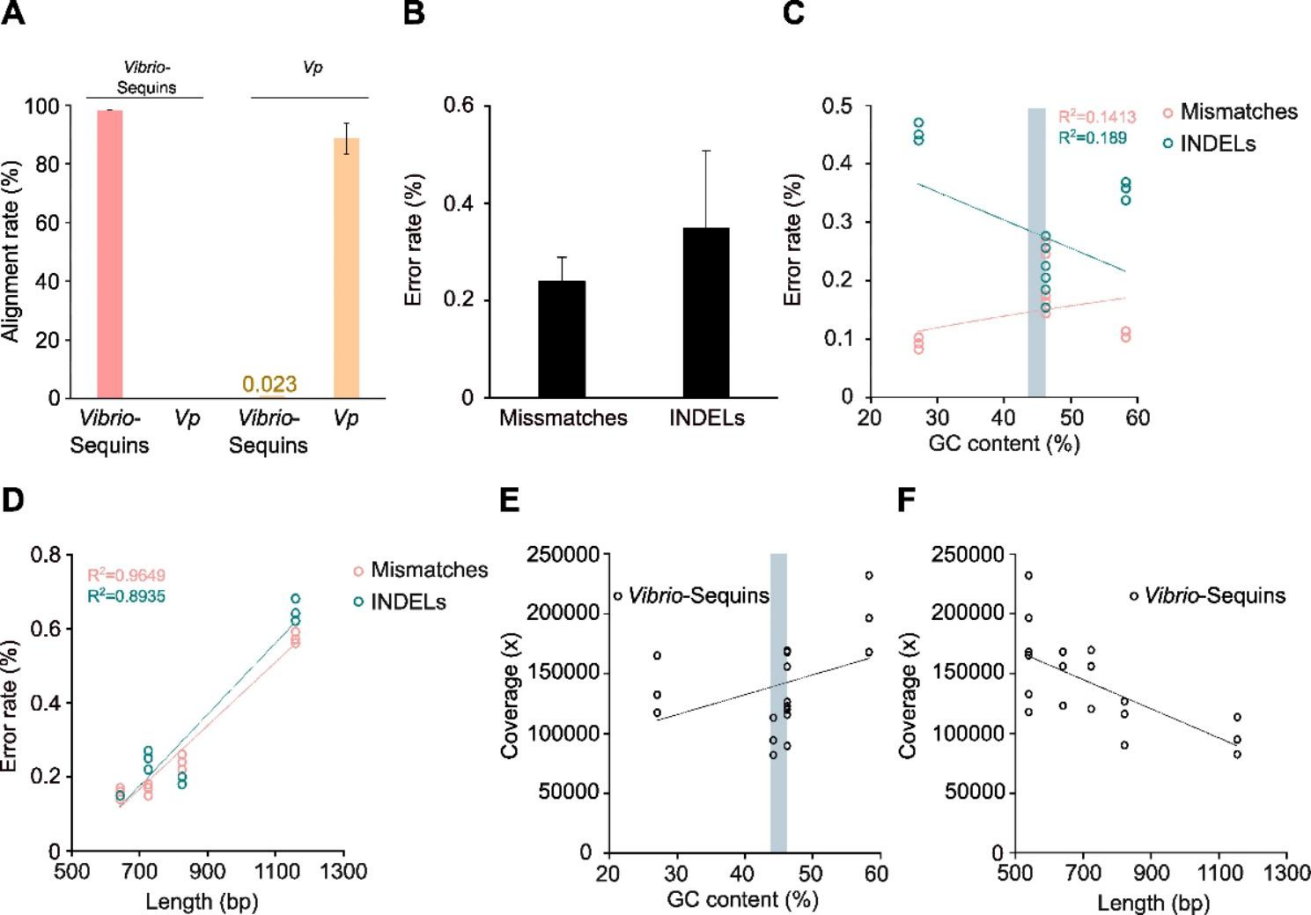
Sequencing performance of *Vibrio-*Sequins. **(A)** Mappability of *Vibrio-*Sequins and *V. parahaemolyticus* reads including cross-alignments was compared by mapping reads to a combined genome index comprising the six *Vibrio-*Sequin sequences and the *V. parahaemolyticus* genome. Data shown for *Vibrio-*Sequins are derived from reads of samples S1-S3, S25-S27 and the *V. parahaemolyticus* reads from samples S30, S32, S33, S41 and S42. Histograms show means ± SD (n ≥ 5). **(B)** Sequencing error rates (mismatches and indels) for *Vibrio-*Sequins. Histograms show means ± SD (n = 6). **(C)** Effect of GC-content on sequencing error rates (mismatches and indels) for *Vibrio-*Sequins. Scatter plots show rate of mismatches (red) and indels (petrol) against the respective GC-content (%). Grey bar indicates average GC-contents of *Vibrio* genomes. **(D)** Effect of length on sequencing error rates (mismatches and indels) for *Vibrio-*Sequins. Scatter plots show rate of mismatches (red) and indels (petrol) against the respective length (bp). **(E)** GC-content-based coverage-bias for *Vibrio-*Sequins. Scatter plots show average coverage against the respective GC-content (%). Grey bar indicates average GC-contents of *Vibrio* genomes. **(F)** Length-based coverage-bias for *Vibrio-*Sequins. Scatter plots show average coverage against the respective length (bp). **(B-F)** Reads are derived from sequencing of pure standards (S1-S3 and S25-S27). **(C-F)** Depicted are the individual replicates for each of the six standards

We further used the results of the sequencing of the pure, undiluted *Vibrio-*Sequin 1:1:1 mixes to investigate mappability and error rates for the standards (Fig. 3A-F). When mapping the reads of the pure standard mixtures to a combined index containing the *Vibrio-*Sequin sequences and the *V. parahaemolyticus* reference genome, we observed only a very small fraction of reads (0.023%; Fig. 3A) that aligned erroneously to the *V. parahaemolyticus* genome sequence, while 99.74% of reads were mapped to *Vibrio-*Sequin sequences, demonstrating excellent mappability. The tiny fraction of *V. parahaemolyticus* mapped reads is most likely the result of contamination derived from sample preparation. In contrast, no reads of *V. parahaemolyticus* samples (S30, S32, S33, S41, S42; additional file 2) mapped to *Vibrio-*Sequin sequences (Fig. 3A). The mappability of *V. parahaemolyticus* was slightly reduced compared to the standards with 89.95% (Fig. 3A), which is likely explained by the sample composition/quality and possibly a novel strain of the sequenced *V. parahaemolyticus* and therefore not perfectly matching reference genome sequence. The percentages of sequencing-derived errors (mismatches and indels) were within the range of previously reported values for the Illumina Miseq system [38] (Fig. 3B). Furthermore, *Vibrio-*Sequin pure, undiluted sequencing was also utilised to assess the impacts of both GC-content and fragment length on sequencing error rates (Fig. 3C-D) and coverage (Fig. 3E-F). While there was no obvious linear relationship visible from our data between error rate and GC-content (Fig. 3C), neither for mismatches nor for indels, we observed an elevated error rate with increasing fragment length of the standard (Fig. 3D). For the GC-content, it rather appeared that the error rate was slightly lower at both extremes - low and high GC-content - compared to average GC-contents of ~ 50% (Fig. 3C). Conversely, we noticed a slight GC-bias in terms of coverage, with elevated coverage at higher GC-contents (Fig. 3E) likely caused by the amplification step during library preparation. In terms of length-bias, we observed a clear negative relationship between fragment length and coverage (Fig. 3F).

### Normalizing coverage and quantifying ***Vibrio-DNA using Vibrio-***Sequins

**Fig. 4:**
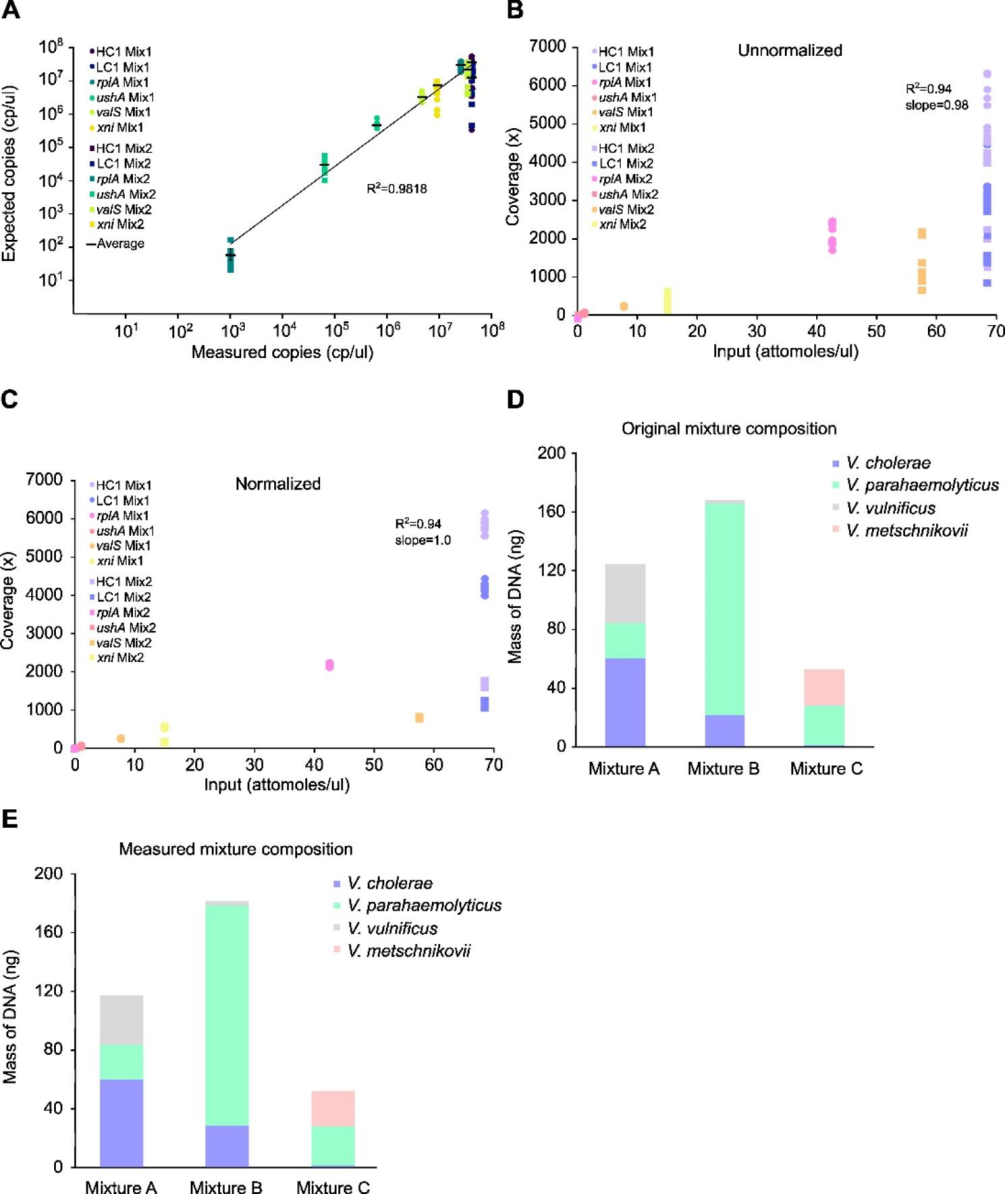
Normalization and quantification using *Vibrio-*Sequins. **(A)** Scatter plot showing the linearity of expected copy number concentrations (cp/µl) vs. measured copy number concentrations (cp/µl) of *Vibrio-*Sequins in individual DNA libraries (S28-S47). Expected copies refer to the copy numbers calculated for each standard after spiking to the samples. These numbers are based on the dPCR-quantified stock solutions of the *Vibrio-*Sequins. Measured copy numbers refer to the dPCR-measured copy numbers after samples have undergone library preparation. Shown are copy numbers (cp/µl) of individual replicates for each of the six standards HC1 (plume), LC1 (dark blue), *rplA* (petrol), *ushA* (green), *valS* (light green) and *xni* (yellow) for both *Vibrio-*Sequin mixes (1 = circles and 2 = squares) as well as the means (black) ± coefficients of variation (%CV; n = 10). **(B)** Quantitative accuracy of *Vibrio-*Sequin sequencing within individual DNA libraries (S28-S47). Scatter plots show the unnormalized coverage of individual replicates for each of the six standards HC1 (lila), LC1 (violet), *rplA* (pink), *ushA* (red), *valS* (orange) and *xni* (yellow) for both *Vibrio-*Sequin mixes (1 = circles and 2 = squares) against the input concentration of the *Vibrio-*Sequins (attomoles/µl). **(C)** Quantitative accuracy of *Vibrio-*Sequin sequencing within individual DNA libraries (S28-S47). Scatter plots show the normalized coverage of individual replicates for each of the six standards HC1 (lila), LC1 (violet), *rplA* (pink), *ushA* (red), *valS* (orange) and *xni* (yellow) for both *Vibrio-*Sequin mixes (1 = circles and 2 = squares) against the input concentration of the *Vibrio-*Sequins (attomoles/µl). Normalization occurred through subsampling reads to the lowest coverage of *Vibrio-*Sequins within a DNA library. **(D)** Three different *Vibrio* mixtures (A, B and C) were assembled from Qubit-quantified DNA, comprising different amounts of DNA from the *Vibrio* species *V. cholerae, V. parahaemolyticus, V. vulnificus* and *V. metschnikovii* and spiked with either *Vibrio*-Sequin mix 1 (Mixtures A and B) or *Vibrio-*Sequin mix 2 (Mixture C) at 2% fractional abundance. **(E)** Quantification of *Vibrio*-derived DNA using *Vibrio-*Sequins in the three *Vibrio* Mixtures (A, B and C) employing the method developed in [23]. dPCR-quantified copy number concentrations (cp/µl) of individual *Vibrio-*Sequins within the DNA libraries (S28, S29 and S38 = *Vibrio* Mixtures A-C) were used for the quantification

In order to quantify *Vibrio*-DNA accompanying the spiked mixes of DNA standards in individual DNA libraries, we employed a method that involves the absolute copy number (cp/µl) quantification of individual *Vibrio-*Sequins in sequencing samples *via* dPCR. Therefore, DNA libraries were diluted to dPCR-quantifiable levels of copies (below 50,000 cp/µl) immediately after enzymatic library preparation and the three validated duplex dPCR methods to quantify *Vibrio-*Sequins were applied. In Fig. 4A, a linear regression of the relationship between the expected and dPCR-measured concentrations (cp/µl) of the DNA standards is given. Shown are *Vibrio-*Sequin concentrations for both standard mixes (1 and 2), which were spiked to 10 samples each and the average measured concentrations over all replicates (cp/µl; black lines; Fig. 4A). The expected concentrations were calculated based on the dPCR-quantified stock solutions of the individual *Vibrio-*Sequins used to generate the *Vibrio-*Sequin mixes. Similar to the earlier discussed differences in *Vibrio-*Sequin coverage (Fig. 2E-F), there were evident differences observed among the measured copies in the individual libraries as seen by the scattering of values (Fig. 4A). This is likely caused by sample composition and quality, ultimately affecting library preparation and the ability to amplify or recover DNA. However, overall the expected and measured copy numbers (averages) correlated strongly as indicated by the R^2^ value of 0.9818 and the slope of 1.01 (Fig. 4A).

Like in other studies employing artificial DNA standards [12, 18, 19, 21, 22, 39], we also observed a strong linear relationship between sequencing coverage and the estimated input concentration (converted to attomoles/µl) for the *Vibrio-*Sequins (R^2^ = 0.94; Fig. 4B). This correlation improved when studying the two standard mixes individually (mix 1: R^2^ = 0.9603 and mix 2: R^2^ = 0.9616; additional Fig. S6A-B). In Fig. 4B, unnormalized values of the coverages of individual standards for both *Vibrio-*Sequin mixes are indicated. Plotting the coverage of *Vibrio-*Sequins versus their input concentration allowed the determination of the LOD as outlined in [20]. In the case of *Vibrio-*Sequins, the LOD was found to be larger than 0.0017 attomoles/µl input, which resulted in a coverage above a single read. As anticipated, the 10 replicates, which were spiked with the same amounts of *Vibrio-*Sequins (either mix 1 or 2), displayed a range of different coverages for the same standard. The scattering of the replicates was even more evident, if the coverage was plotted against the actual dPCR-measured input concentration (attomoles/µl; additional Fig. S6C). The observed scattering aligns with the previously mentioned differences among the DNA libraries due to technical variations that accumulated during library preparation and sequencing. Interestingly, HC1 and LC1 standards were covered very differently (Fig. 4B) although the same amounts of standards were spiked and this in both *Vibrio-*Sequin mixes, suggesting sequencing-related technical issues as the dPCR-measured concentrations of both standards were comparable (Fig. 4A). Taken together, our data clearly demonstrate the need for intersample normalization to counteract sample-specific biases arising from library preparation and sequencing. To do so, we utilised the coverage of the individual *Vibrio-*Sequins in each of the 10 replicates to calculate the differences between the lowest coverage found among the replicates and the values computed for the remaining nine replicates. This calculated factor difference was then used to subsample the other nine replicates to the same coverage as found in the replicate with the lowest coverage (see methods for details). As shown in Fig. 4C, normalizing the sample coverages using *Vibrio-*Sequins, reduced the variability among the replicates significantly, which is further supported by the corresponding statistics given in additional Fig. S7. Our data clearly demonstrate the usefulness of our standards for reducing differences among DNA libraries due to technical effects, preserving biological differences for further analyses.

Previous studies have demonstrated the potential of artificial DNA standards in quantifying DNA *via* NGS [18, 23, 39, 40]. Here, we modified the quantitative metagenomics approach described in [23] by adding the absolute quantification of the artificial standards in the individual DNA libraries by dPCR. To demonstrate the quantitative power of our method, we assembled and sequenced three different mixtures of *Vibrio*-DNA (A, B and C; samples S28, S29 and S38; for details see additional file 7), each containing known amounts of gDNA (Qubit-quantified) of *V. cholerae, V. parahaemolyticus* and *V. vulnificus* (A, B) and *V. metschnikovii* in addition to the three other *Vibrio*-DNAs (C). As with the other samples, *Vibrio-*Sequins were spiked to the three mixtures in 2% fractional abundance before library preparation and sequencing. Figure 4D shows the compositions of the three mixtures based on the Qubit quantifications of the respective gDNA stock solutions, whereas Fig. 4E shows the quantified amounts of each *Vibrio*-DNA in the mixtures based on our modified quantitative metagenomic approach. For example, *Vibrio* mixture A contained 59.85 ng *V. cholerae* DNA, 24 ng *V. parahaemolyticus* DNA and 40.1 ng *V. vulnificus* DNA (Fig. 4D and Additional File 3). Applying our method, we quantified 60.06 ng of *V. cholerae* DNA, 23.7 ng of *V. parahaemolyticus* DNA and 33.09 ng of *V. vulnificus* DNA (Fig. 4E). It is worth noting that this quantitative approach relies on aligning the sequenced reads to a reference genome. Hence, if the reference genome is not perfect for a certain sample, a reduced amount of DNA will be quantified. This may explain the underestimation of *V. vulnificus* DNA in the *Vibrio* mixture A. Another explanation is that the stock solution containing *V. vulnificus* gDNA contained additional DNA from other sources. In summary, our analyses have shown how a small amount of artificial DNA standards can be used to precisely quantify unknown amounts of DNA in food-derived samples, in this case *Vibrio*-derived DNA.

## Discussion

The genomic profiling and quantification of microbial DNA is increasingly applied in basic research and diagnostics of *Vibrio spp*. [6, 41–47]. One substantial advantage of sequencing-based approaches in comparison to conventional culture-based methods is that they do not require any *a priori* knowledge about the sample. Moreover, it is possible to detect very low amounts of *Vibrio*-derived DNA as well as unculturable strains of *Vibrio spp*. However, despite the indispensable technological benefits of (meta)genomics, the analysis of the resulting data and intersample comparisons remain difficult due to technical variations arising during library preparation, sequencing and alignment [20].

Reference standards cannot only help in assessing the technical variation among individual DNA libraries, but also in quantifying unknown amounts of DNA and microbial abundance analyses [18] and have therefore been increasingly applied in sequencing studies [12, 17, 19, 21, 22, 40, 48]. These methods, however, are not metrologically traceable as they relied so far on the fluorometric quantification of the DNA standards prior to spiking [15, 18, 23, 39].

We designed and characterized synthetic reference standards to accompany microbial DNA samples during quantitative genomics. *Vibrio*-Sequins were specifically generated for the detection and quantification of *Vibrio*-derived DNA, mainly from *V. parahaemolyticus*, *V. cholerae* and *V. vulnificus*. Thus, we used *Vibrio spp*. core gene sequences for the design of *Vibrio*-Sequins, which were then inverted to remove sequence homology to naturally occurring sequences. Our standards cover a range of GC-contents and fragment lengths allowing extensive investigation of sequencing-related biases and errors in distinct DNA libraries (Fig. 3). The six *Vibrio*-Sequins are meant to be applied in combination with six optimized TaqMan assays for their quantification *via* dPCR to enable quantitative genomics that are metrologically traceable to a single DNA copy.

In this study, we validated three duplex dPCR methods to quantify absolute DNA copy numbers (cp/µl) of the reference standards within individual DNA libraries. The quantified amounts of the *Vibrio*-Sequins can then be utilised for the quantification of *Vibrio*-derived gDNA accompanying the standards within DNA libraries. As an integral part of the method validation processes, LODs and LOQs were determined for the individual assays (Fig. 1). In contrast to previous studies utilising more established dPCR technologies than the Stilla Naica crystal dPCR system (e.g., Biorad, Qiagen) [36, 37, 49, 50], we report slightly elevated values for LODs between 10 and 26 cp/µl and consequently for LOQs between 23 and 124 cp/µl (Fig. 1). This is likely explained by (1) the technology and (2) the sample type, as other studies have utilised matrix-free plasmid-DNA for the validation [36, 50], whereas we used PCR amplicons of the standards in a matrix of *Vibrio*-DNA to mimic “target” DNA libraries consisting of *Vibrio*-DNA extracts. Another limitation of our dPCR method validation is the exclusion of the uncertainty associated to the droplet volume, which has been described as an integral part of the overall MU of such methods [36, 51]. Neither the manufacturer nor other studies have, to our knowledge, investigated the uncertainty associated to the droplet volume of the Stilla Naica system, which we are therefore unable to include into our MU estimations. Future efforts should be made towards reliably estimating the droplet volume uncertainty associated to Stilla’s Naica system. In addition, we observed significant discrepancies between Qubit and dPCR quantifications for the three standards *ushA*, HC1 and LC1, in particular for the latter two (Fig. 1). One explanation might be that the estimation of copy numbers in the stock solution based on Qubit measurements were inaccurate for these two standards. Possibly, there was a bias occurring due to the altered GC-contents of these two standards. Another explanation might be that an overamplification of HC1 and LC1 occurred during PCR due to their reduced or elevated GC-contents. Furthermore, it is also conceivable that the amount of intact double-stranded DNA, which is measurable by the Qubit, and the amount of amplifiable DNA that can get quantified by dPCR, differed substantially in the case of HC1 and LC1.

The sequencing of environmental samples can be affected by a variety of technical variables, such as the method of library preparation [52], the sequencing technology [53], the composition of the samples and whether or not enzymatic inhibitors are present in the reactions. Here, we showed that *Vibrio*-Sequins can be employed to determine and mitigate a variety of error sources (Fig. 3) and differences among DNA libraries (Fig. 2). *Vibrio*-Sequins can be applied to normalize between different library preparation and sequencing methods, between experiments and runs, and between individual DNA libraries (Fig. 4), significantly reducing variability caused by technical variables, while preserving biologically meaningful differences. Besides their quality in normalization, *Vibrio*-Sequins represent an essential tool in quantifying DNA derived from *Vibrio spp*. (Fig. 4). We have shown that by coupling NGS to the SI-traceable reference method dPCR and through use of the *Vibrio*-Sequins, we achieved the precise quantification of several *Vibrio*-DNAs in a mixture (Fig. 4). Hence, a possible application of our approach is the quantification of unknown amounts of DNA in metagenomic samples, enabling the detection of trace-amounts of *Vibrio*-DNA. Implementation of our *Vibrio*-Sequins and coupling of NGS to dPCR will increase the accuracy of existing metagenome sequencing methodologies. One limitation to this approach, however, is that it relies on the mapping of sequencing reads to reference genome sequences, which can cause reduced quantification accuracy through non-specific mapping and reduced mappability. An obvious issue related to alignments is the choice of the reference genome sequences, which can directly reduce the amount of quantified DNA if not matching perfectly with the sequenced reads. One attempt to circumvent such problems is to apply bioinformatics tools to predict *Vibrio* strains from raw sequencing reads, such as Vicpred for *V. cholerae* strains [54], prior to NGS-based DNA quantification.

## Conclusion

In summary, we have demonstrated how the coupling of NGS and dPCR *via* reference standards can advance existing quantitative NGS-based approaches in terms of precision and accuracy. We provide avenues to metrological traceability of quantitative sequencing, opening up new directions for the integration of measurement science into NGS-based approaches. Future work should be guided towards understanding sources of uncertainty related to genomic analyses in particular those resulting in significant variability in the resulting data and a lack of comparability. The herein developed *Vibrio*-Sequins and similar synthetic reference standards will be useful for exactly such undertakings.

## Methods

### Design of ***Vibrio-***Sequins

Artificial *Vibrio*-like core gene sequences present in the three *Vibrio* species of interest (*V. cholerae, V. parahaemolyticus* & *V. vulnificus*) were selected from the *V. cholerae* core genome multilocus sequence typing (cgMLST) dataset hosted at public database for molecular typing and microbial genome diversity (pubMLST) [55]. The database search revealed several homologous candidate core genes of high sequence similarity.

Four genes (*rplA, ushA, valS* and *xni*; additional Table 1) with the highest sequence similarity were chosen for the design of *Vibrio-*Sequins. *Vibrio-*Sequins of lengths between 540 and 1156 bp were designed from the *V. parahaemolyticu*s (RIMD 2,210,633; GCA_000196095.1 ASM19609v1) genome sequence. These four genes cover precisely the average GC-content of *Vibrio* genomes (44–47%) [43, 46, 56]. Two additional sequences were designed to cover lower (LC1; 27%) and higher (HC1; 58%) GC-contents than the average *Vibrio* genome. To select candidate gene sequences, the *V. parahaemolyticu*s reference genome was partitioned *in silico* into 500 bp-sized non-overlapping windows using the BEDTools (v2.27.1) [57] function *makewindows* and subsequently these windows were screened for low and high GC-contents using the *nuc* function. To reduce potentially erroneous selection of windows with generally low sequencing coverage, selected windows were confirmed against in-house sequencing data generated from *V. parahaemolyticus*.

While maintaining nucleotide composition exactly as observed in *V. parahaemolyticus*, inversion of the sequences of the six *Vibrio-*Sequins assured sequence uniqueness, while still maintaining GC-content, fragment length and DNA motifs, as described previously [12, 19–22]. To exclude potential similarity to naturally occurring sequences, all inverted *Vibrio-*Sequins were queried against the whole BLAST nt database as available on 13.09.22. No significant similarities were found to sequences included in the nt database. Additional information on the sequences of *Vibrio-*Sequins is provided in additional file 1.

To enable full-length amplification of the individual standards, each sequence was flanked by the matching sequences for T7 (forward) and T3 (reverse) primer-based amplification (additional file 1 & additional Table 2). Subsequently, *Vibrio-*Sequins were synthesized individually by Genescript (Genescript Biotech Corp, USA) and delivered in pUC57 plasmids. Using a high-fidelity Phusion polymerase (New England Biolabs, USA) according to manufacturer’s protocol, full-length amplicons of the six individual standards were generated. Amplification products were purified using the Qiagen PCR purification kit (Qiagen, Netherlands) and the individual sequences were confirmed by Sanger sequencing (Microsynth, Switzerland). Aliquots of thousand-fold dilutions of the individual PCR-amplicons were stored at -20 °C.

### Design and optimization of TaqMan assays

To enable absolute quantification of the individual *Vibrio-*Sequin standards present in each DNA library by crystal digital PCR (Stilla Technologies – Naica system; henceforth dPCR) [58], we firstly developed suitable TaqMan assays for each *Vibrio-*Sequin. The term “assay” refers to a specific combination of forward and reverse PCR primers and a single combined quencher-fluorophore PCR probe. Using the real-time PCR (TaqMan) primer and probes design tool (Genescript; https://www.genscript.com/tools/real-time-pcr-taqman-primer-design-tool) or alternatively Primer3 (https://primer3.org/), primers and probes for each standard were designed following the “qPCR guide” by Eurogentec (https://www.eurogentec.com/assets/7787b90a-cf84-4acf-8e37-f36487b9f091/guide-en-qpcr-guide.pdf). Subsequently, primers and probes were synthesized by Microsynth (Switzerland). Upon arrival, lyophilised primers and probes were eluted in nuclease-free H_2_O (Thermofisher Scientific, USA) to generate 100 µM solutions and stored at -20 °C. From these stock solutions, aliquots of 10 µM working solutions were prepared and stored likewise at -20 °C. Primer specificity was tested by conventional PCR utilizing the above-described plasmids (0.1 ng/ul concentrations) containing single *Vibrio-*Sequins (additional Fig S1) in a *Vibrio*-DNA matrix. Individual TaqMan assays were optimised by quantitative PCR (qPCR) in singleplex mode prior to their validation by dPCR. We therefore compared amplifications at 13 different combinations of forward and reverse primer concentrations (50/50 nM, 50/100 nM, 100/50 nM, 100/100 nM, 100/300 nM, 300/100 nM, 300/300 nM, 300/600 nM, 600/300 nM, 600/600 nM, 600/900 nM, 900/600 nM and 900/900 nM; additional Fig S2) for each individual *Vibrio-*Sequin at otherwise consistent conditions (e.g., probe concentration of 150 nM, PCR cycling, master mix) using the PCR-amplicons of the standards at a ~ 20000 cp/ul concentration. Amplification of individual standards were examined by qPCR using the Perfecta Multiplex qPCR ToughMix (5X) according to manufacturer’s recommendations (Quantabio, USA) and the MIC qPCR system (Bio Molecular Systems, Australia). qPCR was performed in triplicates and cycling was as follows: initial 3 min at 95 °C followed by 15 s at 95 °C and 15 s at 60 °C for 45 cycles. In each run, no template controls (NTCs) were included along with negative controls (*Vibrio*-DNA). Thresholds were set manually within the exponential part of the amplification curves. Following primer optimizations, PCR efficiencies were determined as described in [59], using 6 serial dilutions (10-fold) of the standard’s PCR amplicons starting from a 100000-fold dilution of the purified PCR products. PCR conditions and PCR mix compositions were applied as described before. qPCR was performed in triplicates. Optimised primer concentrations for each standard are depicted in additional Fig S2. Primers, probes and PCR efficiencies are listed in additional file 3. Amplicon lengths and locations are depicted in additional file 8.

### Validation of dPCR methods for ***Vibrio-***Sequins

The validations of dPCR methods for the individual standards were performed according to the ISO Guide 20395 [34]. In brief, for each assay we analysed 10 different concentration levels of individual *Vibrio-*Sequin PCR-amplicons with 10 replicates at each concentration level. Replicates were split over 3 different runs to allow determination of inter-run variation [36]. DNA concentrations of the thousand-fold solutions of the individual standards (see previous section) were quantified using the HS dsDNA Qubit Assay on a Qubit 4.0 (Life Technologies, USA) and were sequentially diluted to generate 10 dilutions covering an estimated range of 3–50000 cp/µl. *Vibrio*-DNA (see section “sample collection, cultivation and DNA extraction”) was used as a matrix for the dilutions of the standards. The PCR mix comprised the Perfecta Multiplex qPCR ToughMix (5X), primers and probes as optimized in the qPCR (see previous section), nuclease-free H_2_O (Thermofisher Scientific, USA) and the DNA sample. Nucleotide sequences of primers and probes are listed in additional Table 2 and optimized primer concentrations are depicted in additional Fig S2. Probe concentrations were set to 150 nM. In each run, one NTC was included. 25 µl of the PCR mixes were transferred onto Sapphire chips (Stilla Technologies, France) and the chips were loaded onto the Geode automated droplet generator and thermocycler (Stilla Technologies, France) according to the instructions of the manufacturer to partition the samples at 40 °C and 950 mbar for 12 min. PCR amplification was performed at universal conditions: 7 min at 95 °C, followed by 45 cycles of 15 s at 95 °C and 60 s at 60 °C. Air pressure was released to ambient conditions at 95 °C for 10 min. Ramp rate was set to 2 °C/s. After PCR amplification, the chips were transferred to the Prism3 multi-color fluorescence imager (Stilla Technologies, France) for scanning. Data analysis was performed using the Crystal Reader and Crystal Miner software (Still Technologies, France). dPCR method validations were performed in duplex formats – HC1 (Cy5 – exposure time 50 ms) and LC1 (FAM – exposure time 65 ms), *ushA* (Cy5.5 – exposure time 50 ms) and *valS* (Cy3 – exposure time 250 ms), *rplA* (FAM – exposure time 65 ms) and *xni* (Cy5 – exposure time 50 ms). A partition volume of 0.53 nl was assumed for the DNA copy number calculations according to batch-information provided by the chip manufacturer. Thresholds were applied as suggested by the Crystal Miner software (Stilla Technologies, France). Copy number calculations including coefficients of variation (%CV) were performed according to the ISO Guide 20395 [34]. The following method performance parameters were assessed during validation: specificity, precision, repeatability, limits of quantification and detection (LOQ and LOD), linearity and finally the expanded measurement uncertainty (MU), according to the ISO Guide 20395 [34]. The dMIQE20 checklist [35] can be found in additional file 2.

### Preparation of ***Vibrio-***Sequin mixtures for spiking

To prepare *Vibrio-*Sequin mixes 1 and 2 for the spiking of samples, thousand-fold solutions of the individual PCR-amplicon standards have been dPCR-quantified using the validated dPCR methods described in the section “Validation of dPCR methods for each standard”. Quantification was done in triplicates, one NTC was included in each run. Using the dPCR-derived DNA copy numbers for the six *Vibrio-*Sequins, two mixes were prepared containing all six standards covering a ~ 3.3 × 10^5^-fold concentration range over both mixes, enabling the estimation of the LOD for sequencing. Individual sequins were pooled according to the table provided in additional file 2. Mixture stocks were prepared as single-use aliquots and stored at -20 °C.

### Sample collection, cultivation and DNA extraction

In order to get *Vibrio*-derived DNA for the sequencing and dPCR validation experiments, fresh and refrigerated fish and seafood samples (tuna, salmon, shrimps) were purchased from local retail markets and fish shops in Bern, between February and September 2022. Additionally, *Vibrio*-DNA was derived from the following reference strains; *Vibrio parahaemolyticus* (NCTC 10,885, NCTC 11,058), *V. vulnificus* (DSMZ 10,143) and *V. cholerae* (NCTC 8042, NCTC 11,348). To isolate *Vibrio* gDNA, the ISO method 21872-1:2017 [9] was applied, with slight modifications. In brief, 25 g of sample material was homogenized in 225 ml of alkaline saline peptone water (ASPW, Oxoid, CM1117B) by using a stomacher blender for 1 min. The homogenate was incubated for 6 h at 37 °C. Then 1 ml culture of the first enrichment step was transferred to 9 ml of fresh ASPW in a reaction tube and incubated for 18 h at 37 °C. In parallel, a second aliquot of 1 ml culture in 9 ml of ASPW was incubated for 18 h at an increased temperature of 41.5 °C. The grown media of the second enrichment step were then streaked on thiosulfate citrate bile and sucrose agar (TCBS, Millipore, 86348), *Vibrio* ChromoSelect agar (Millipore, 92323) and ChromID Vibrio agar (bioMérieux, 43761). The plates were incubated for 24 h at 37 °C. Presumptive colonies were picked, streaked on saline nutrient agar (SNA, Oxoid, CM0003) and incubated for 24 h at 37 °C. Oxidase-positive colonies were identified by using Oxidase strips (Oxoid, MB0266A). Positive clones were resuspended in 300 µl ASPW, centrifuged for 3 min at 20’000 g and the pellet was used for DNA extraction by the DNeasy Blood & Tissue kit (Qiagen, Netherlands) on the QIAcube instrument following manufacturer’s instructions. DNA extracts of isolates were used for species identification by real-time qPCR methods prior to applying the DNA in sequencing experiments. *V. parahaemolyticus* was identified by primers targeting the toxR gene [60], for *V. vulnificus* the cytolysin gene vvhA [61] and for *V. cholerae* the outer membrane protein ompW gene [62].

To investigate the quantitative power of *Vibrio-*Sequins, three *Vibrio* mixtures (A, B and C) were prepared with known DNA concentrations of each *Vibrio* species (Qubit quantified): *V. cholerae, V. parahaemolyticus, V. vulnificus* DNA (A and B), as well as *V. metschnikovii* DNA (C; derived from food sample). DNA concentrations were determined using the HS dsDNA Qubit Assay on a Qubit 4.0 (Life Technologies, USA). Mixture compositions are listed in additional file 3.

### Spiking, library preparation and sequencing

A full list of sequenced samples is provided in additional Table 3. *Vibrio-*Sequin mix 1 was spiked to samples S25-S37, whereas samples S38-S47 were spiked with *Vibrio-*Sequin mix 2. Standards were spiked at 2% fractional abundance. Moreover, *Vibrio-*Sequins were sequenced pure and undiluted in triplicates (S1-S3 & S25-S27) to assess technical variation and sequencing errors. The Illumina DNA Prep Tagmentation Kit (Illumina, USA) was used to prepare DNA libraries according to manufacturer’s protocol with a standard DNA input of ~ 120 ng DNA (except for samples S28, S29, S37, S38, S39 and S46 (additional file 3). DNA libraries were quantified using the HS dsDNA Qubit Assay on a Qubit 4.0 (Life Technologies, USA) and fragment sizes were examined on a 2100 Bioanalyzer using the High Sensitivity DNA Kit (Agilent Technologies, USA). Subsequently, DNA libraries were pooled and denatured to a 16.5 pM combined library and sequenced on a Miseq system using Miseq v2 chemistry (Illumina, USA) with 2 × 201 bp reads. Raw sequencing data are available at NCBI’s Sequence Read Archive under the project number PRJNA914529.

### ***Vibrio-***Sequin quantification in DNA libraries

To determine the absolute DNA copy numbers of each *Vibrio-*Sequin within the individual DNA libraries after the samples have undergone library preparation, individual libraries (unpooled) were diluted with nuclease-free H_2_O (Thermofisher Scientific, USA) to render quantifiable samples for dPCR. Dilutions were prepared according to the spiked copy numbers of *Vibrio-*Sequins prior to library preparation (2% *Vibrio-*Sequin mixtures). Standards were quantified using the validated dPCR methods described in the section “validation of dPCR methods for each standard”. Quantification of each library was done in duplicates, NTCs were included in each run.

### Bioinformatics

FastQC (v0.11.9; http://www.bioinformatics.babraham.ac.uk/projects/fastqc/) was applied for quality control of raw sequencing reads. Reads were quality-trimmed using Trimmomatic (v.039)[63] in paired-end mode with the parameters: Illuminaclip:4:30:10 headcrop:16 crop:196 minlen:185. Prior to mapping, a combined indexed reference genome containing the *Vibrio-*Sequin DNA sequences along with the *Vibrio* genome sequences was generated through concatenation and indexing with BWA index (v0.7.17-r1188) [64]. De-multiplexed FASTQ files were then mapped against the generated index using the aligner BWA MEM (v0.7.17-r1188)[64] in paired-end mode. Average per-base coverage shown in Fig. 2 (pure and undiluted *Vibrio-*Sequins; S1-S3 and S25-S27) was computed from mapped BAM files using the BEDTools (v2.27.1)[57] *genomecov* function with 100 bp excluded from each end of the *Vibrio-*Sequins to avoid edge effects. The percentage of mapped reads of *Vibrio-*Sequins were estimated using Fastqscreen (v0.15.1)[65] with the BWA MEM aligner (v0.7.17-r1188) [64]. Average coverage of individual *Vibrio-*Sequins, alignment and error rates (mismatches and indels) were determined using Qualimap 2 (v2.2.1)[66] on BAM files of samples S1-S3 and S25-S27, containing the undiluted standards, as well as on *V. parahaemolyticus* samples S30, S32, S33, S41, S42 (for alignment of *V. parahaemolyticus*; additional Table 3). Coverage distributions of *Vibrio-*Sequin replicates were visualized with IGV (v.2.13.3) [67]. Average per-base coverage depicted in Fig. 4 (samples S28-S47) was computed from mapped BAM files using the BEDTools (v2.27.1)[57] *genomecov* function with 100 bp excluded from each end of the *Vibrio-Sequins* to avoid edge effects. Normalization of average per-base coverage among the samples (containing the same *Vibrio-*Sequin mix) was achieved through subsampling to the lowest average coverage of *Vibrio-*Sequins among the samples using SAMtools (v1.10)[68] *view –s* function. Quantification of *Vibrio* species in *Vibrio* mixtures (A, B and C) employing *Vibrio-*Sequins followed the method in [23] with some modifications: briefly, the number of sequenced bases was computed from mapped BAM files using SAMtools (v1.10)[68] *stats* function. Number of mapped bases to each standard was estimated with Qualimap 2 (v2.2.1)[66] on BAM files. The concentrations (ng/µl) of individual *Vibrio-*Sequins were determined by dPCR as described in section “*Vibrio-*Sequin quantification in DNA libraries”.

## Electronic supplementary material

Below is the link to the electronic supplementary material.

Additional file 1. Figures S1-S7.

Additional file 2. Table S1. Information on *Vibrio-*Sequins.

Additional file 3. Table S2. Oligonucleotides and probes used in this study.

Additional file 4. Table S3. Sample information.

Additional file 5. dMIQE2020

Additional file 6. Composition of *Vibrio-*Sequin mixes 1 and 2.

Additional file 7. Composition of *Vibrio* mixtures A, B and C.

Additional file 8. Information on amplicon location.

## Data Availability

The datasets supporting the conclusions of this article are included within the article (and its additional files). The generated sequencing datasets are publicly available from the NCBI Sequence Read Archive under the project number PRJNA914529. All other datasets (dPCR, qPCR) are available from the corresponding author upon request.
