# Supplementary material for: Vibrio-Sequins - dPCR-traceable DNA standards for quantitative genomics of Vibrio spp
Source: BMC Genomics. 2023 Jul 4;24:375. doi: 10.1186/s12864-023-09429-8 (PMC10318669; doi:10.1186/s12864-023-09429-8)
Supplement: Supplementary file 1 — Additional file 1. Figures S1-S7. [file 12864_2023_9429_MOESM1_ESM.docx]

**Supplementary Information for Flütsch *et al*., - Additional file 1:**

**
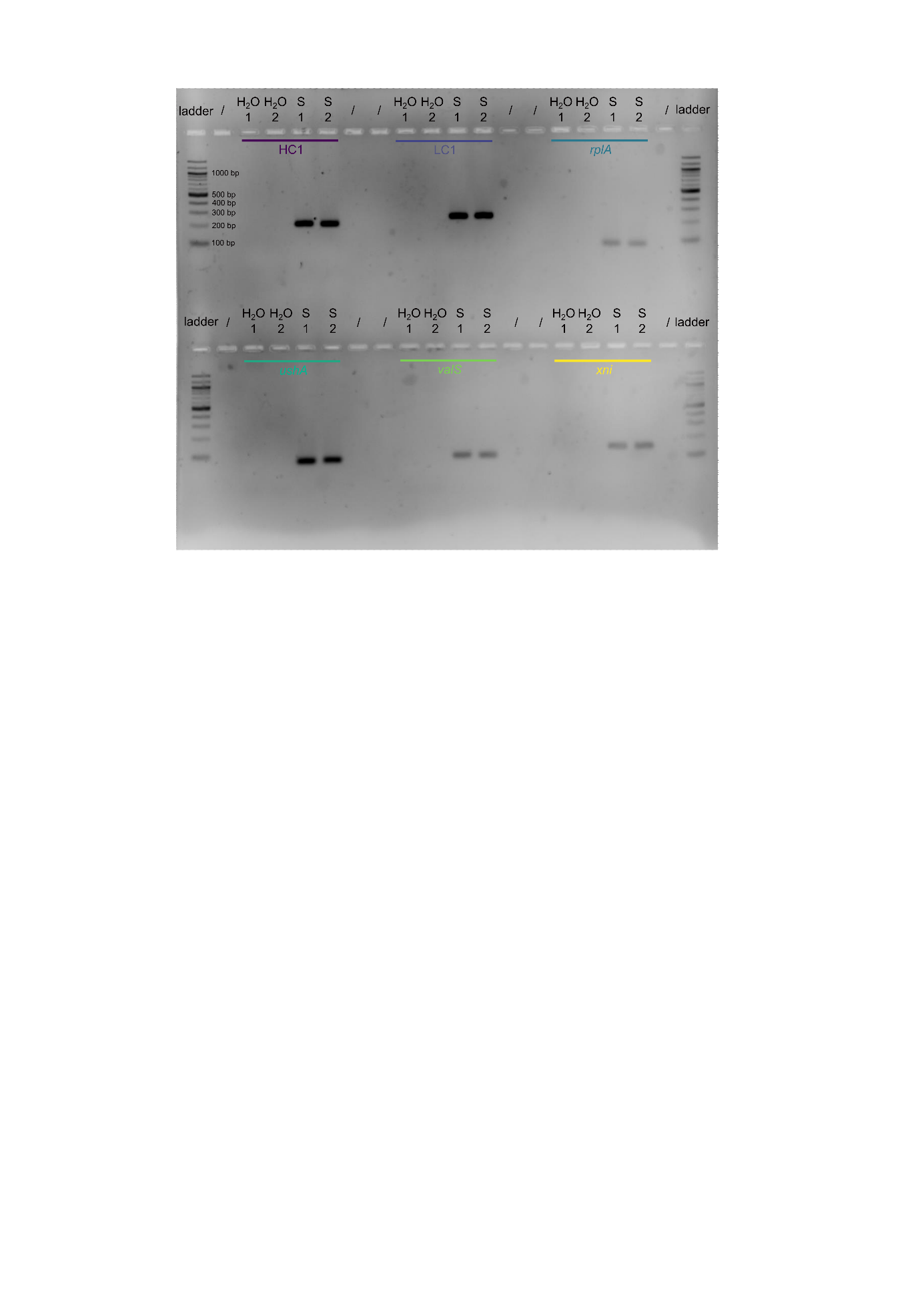
**

**Additional Fig. S1 Primer specificity at 60°C annealing temperature for *Vibrio*-sequin TaqMan assays.** Plasmid DNA (0.1 ng/µl) of the individual *Vibrio-*sequins in a matrix of *Vibrio*-DNA was amplified using the primers listed in additional file 3. S = sample. For each TaqMan assay, PCR products were loaded in the following order: 2 x H_2_O controls, 2 x respective plasmid samples. 100 bp ladder was used.

**
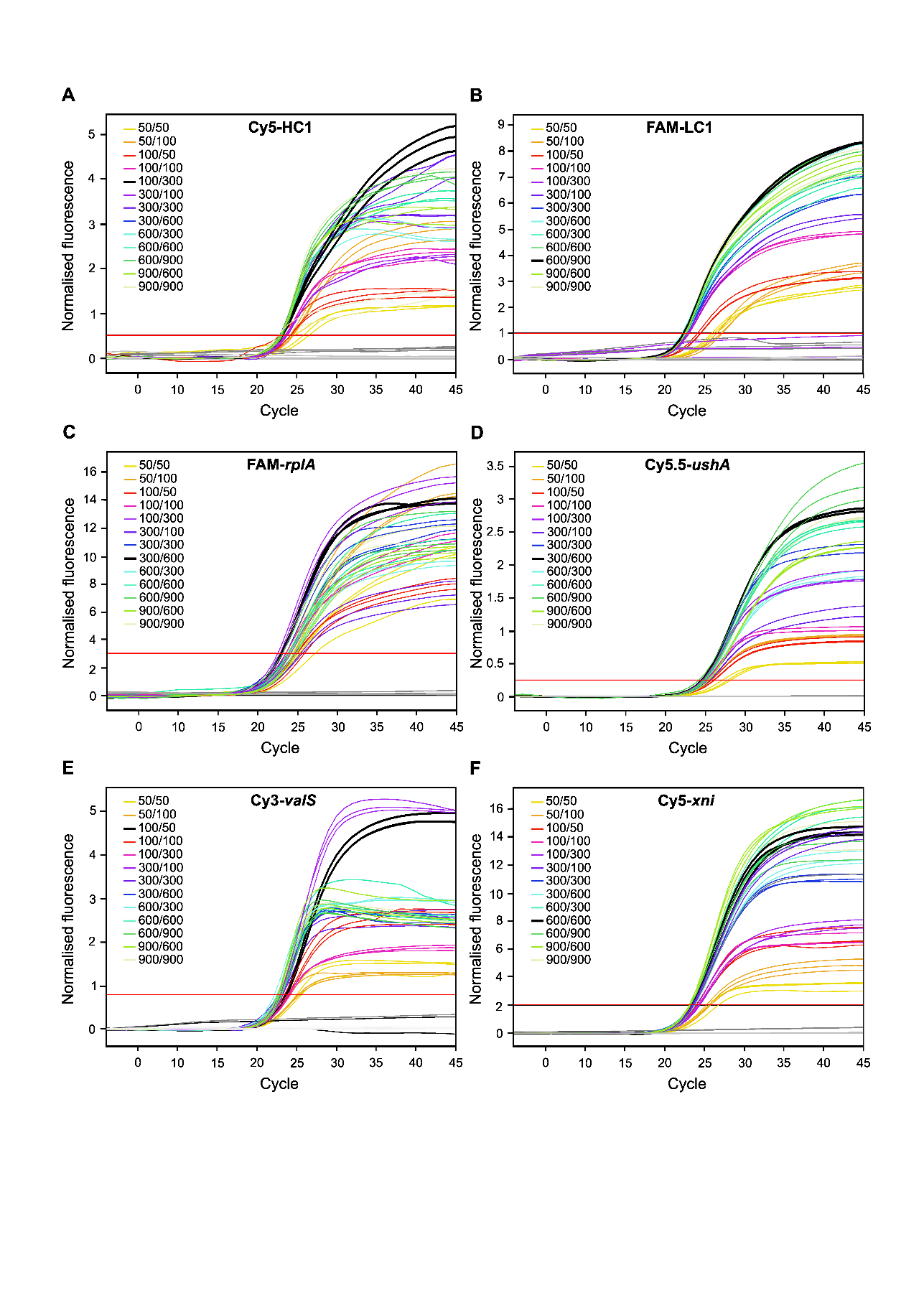
**

**Additional Fig. S2 qPCR-based primer optimization of *Vibrio*-sequin TaqMan assays.** A - F) Amplification curves for the 13 tested primer concentration combinations of the primer optimization matrix for the individual *Vibrio*-sequin TaqMan assays A) HC1, B) LC1, C) *rplA*, D) *ushA*, E) *valS* and F) *xni*. Primers for the individual assays are listed in additional file 3. No amplification was observed for the no template controls (NTCs; light grey) and the negative controls (DNA from non-targeted *Vibrio*-sequin PCR amplicons; dark grey). Primer concentrations are indicated in the same color as the corresponding curves in the amplification plots. Optimal primer concentration for each assay are represented in black. Primer concentrations are given in nM.

**
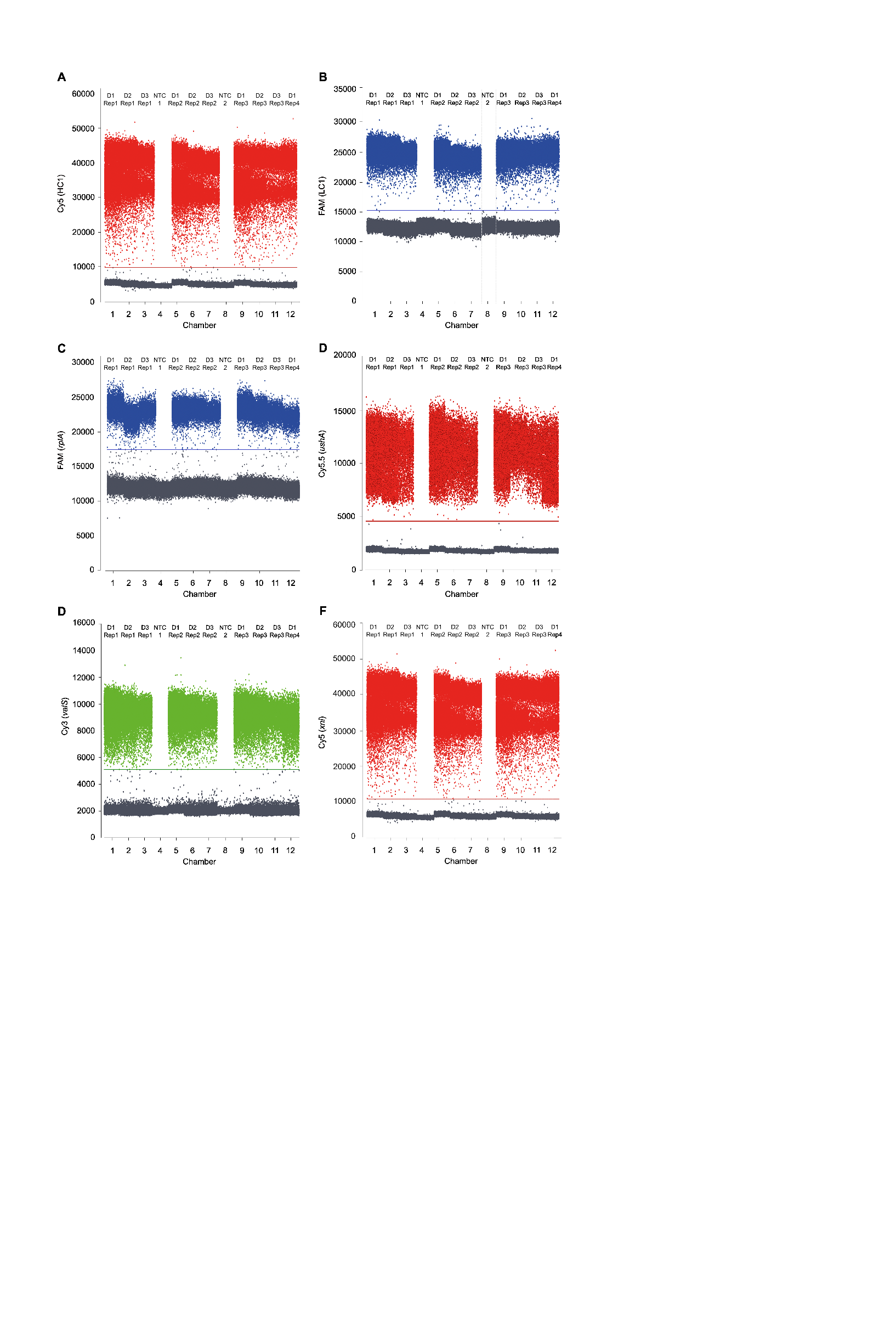
**

**Additional Fig. S3 dPCR traces of *Vibrio*-sequin TaqMan assays.** A – F) Characteristic droplet profiles of crystal dPCR-measured *Vibrio*-sequin PCR amplicons A) HC1, B) LC1, C) *rplA*, D) *ushA*, E) *valS*, F) *xni*, employing the optimized *Vibrio*-sequin TaqMan assays. D1 ~ 50000 cp/µl, D2 ~ 12500 cp/µl, D3 ~ 6250 cp/µl, NTC = no template control = corresponding master mix + H_2_O. Y-axis shows relative fluorescence units. Primers for the individual assays are listed in additional file 3.


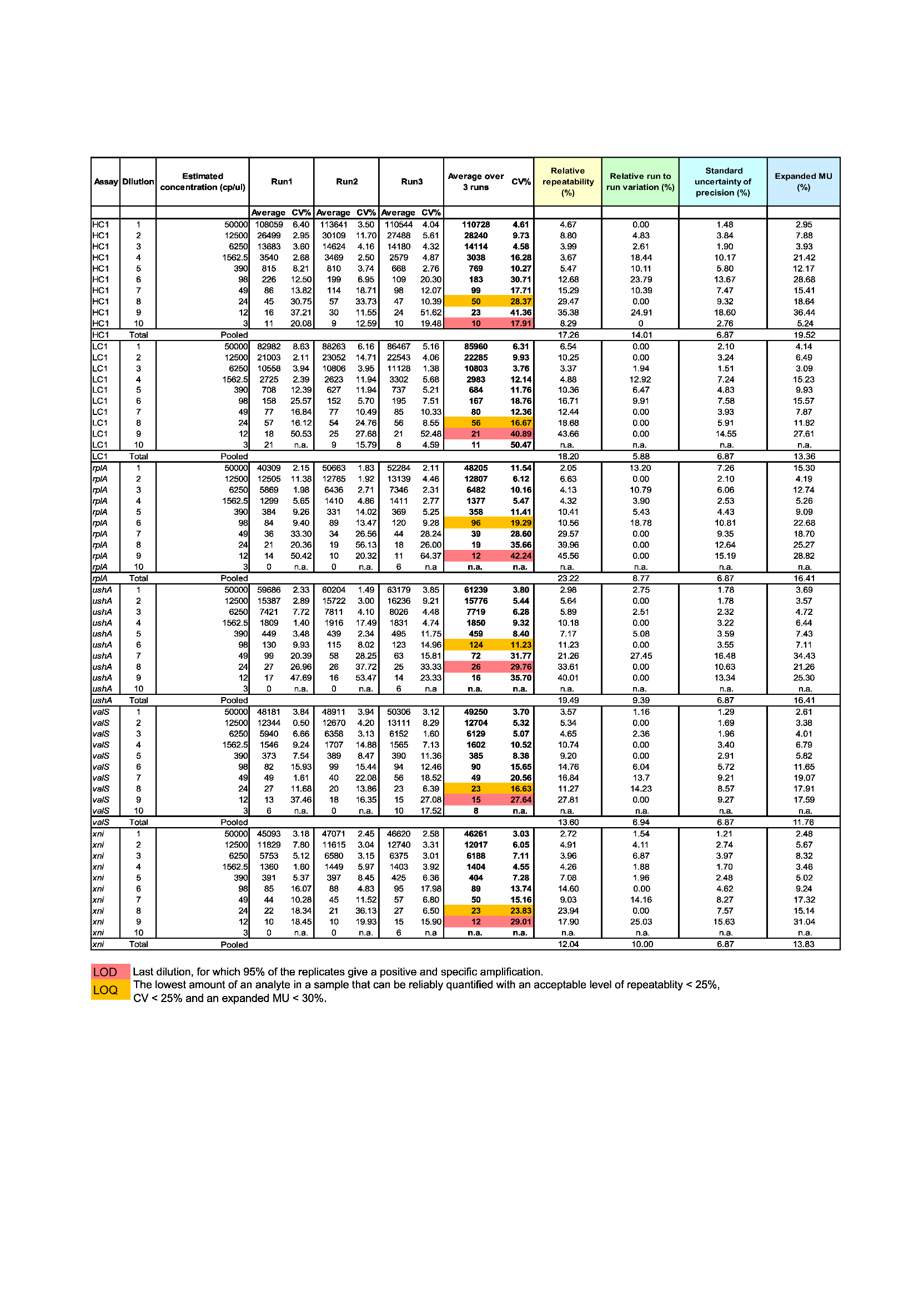


**Additional Fig. S4 dPCR validation study.** Detailed results of the dPCR method validations for each *Vibrio-Sequin* standard. Data shown are the estimated copy number concentrations (cp/µl) from Qubit measurements, the average dPCR-determined copy number concentrations (cp/µl) for each of the three runs along with their coefficients of variation (%CV), the overall average dPCR-determined copy number concentrations (cp/µl) and the overall %CV, calculated relative repeatability (%), relative run-to-run variation (%), relative standard uncertainty related to precision (%) and the MU for *k*=2. In cases were MS_betweenrun_ < MS_withinrun_, the relative run-to-run variation was considered negligible compared the relative repeatability and set to 0.


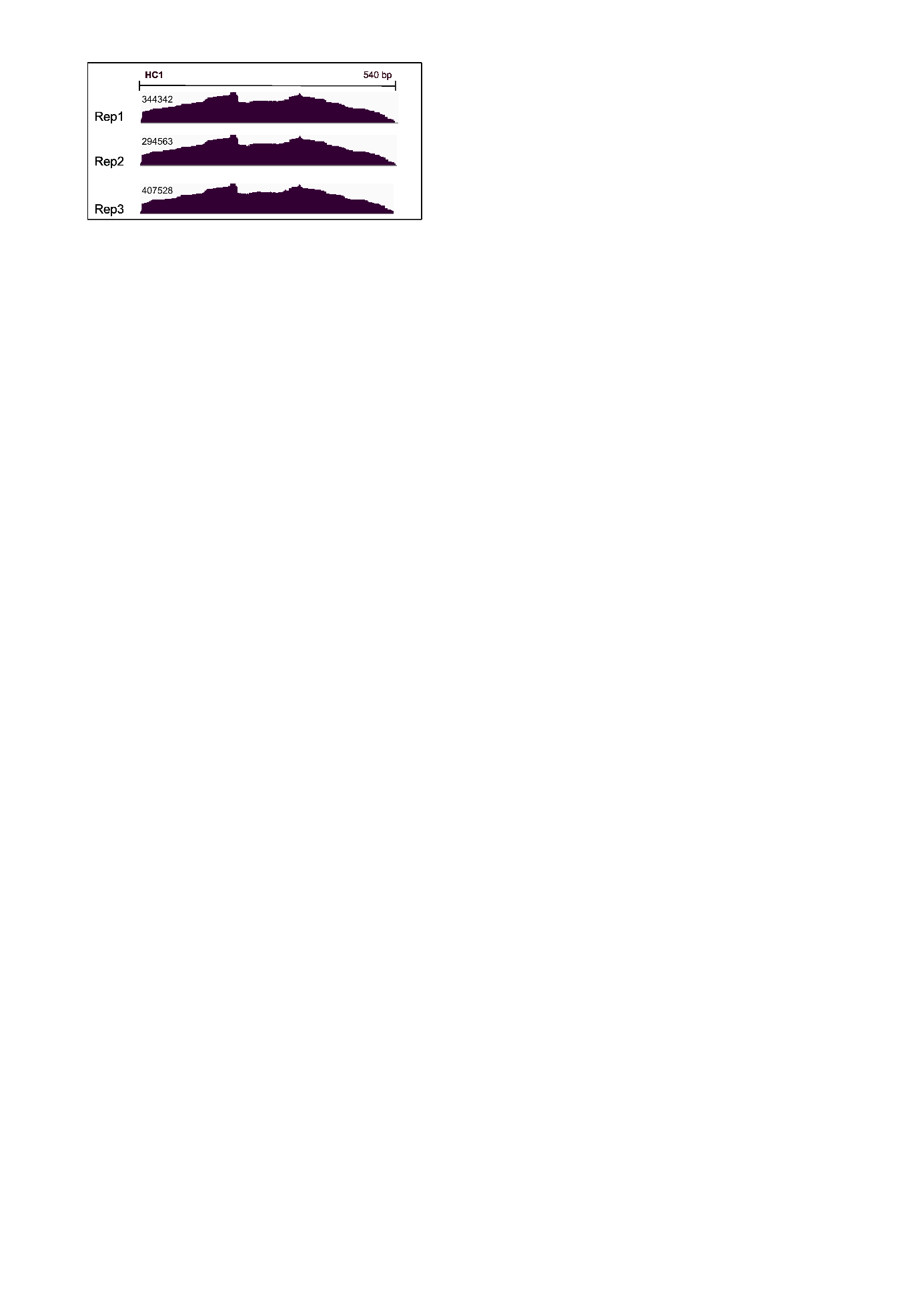


**Additional Fig. S5 Coverage of replicates of sequencing pure and undiluted *Vibrio*-sequin HC1.** IGV-visualized coverage plots across the full-length HC1 sequence (540 bp). Sequencing data are derived from sequencing of samples S1-S3 containing equal amounts of pure *Vibrio*-sequins HC1, LC1 and *ushA* (40 ng each).

**
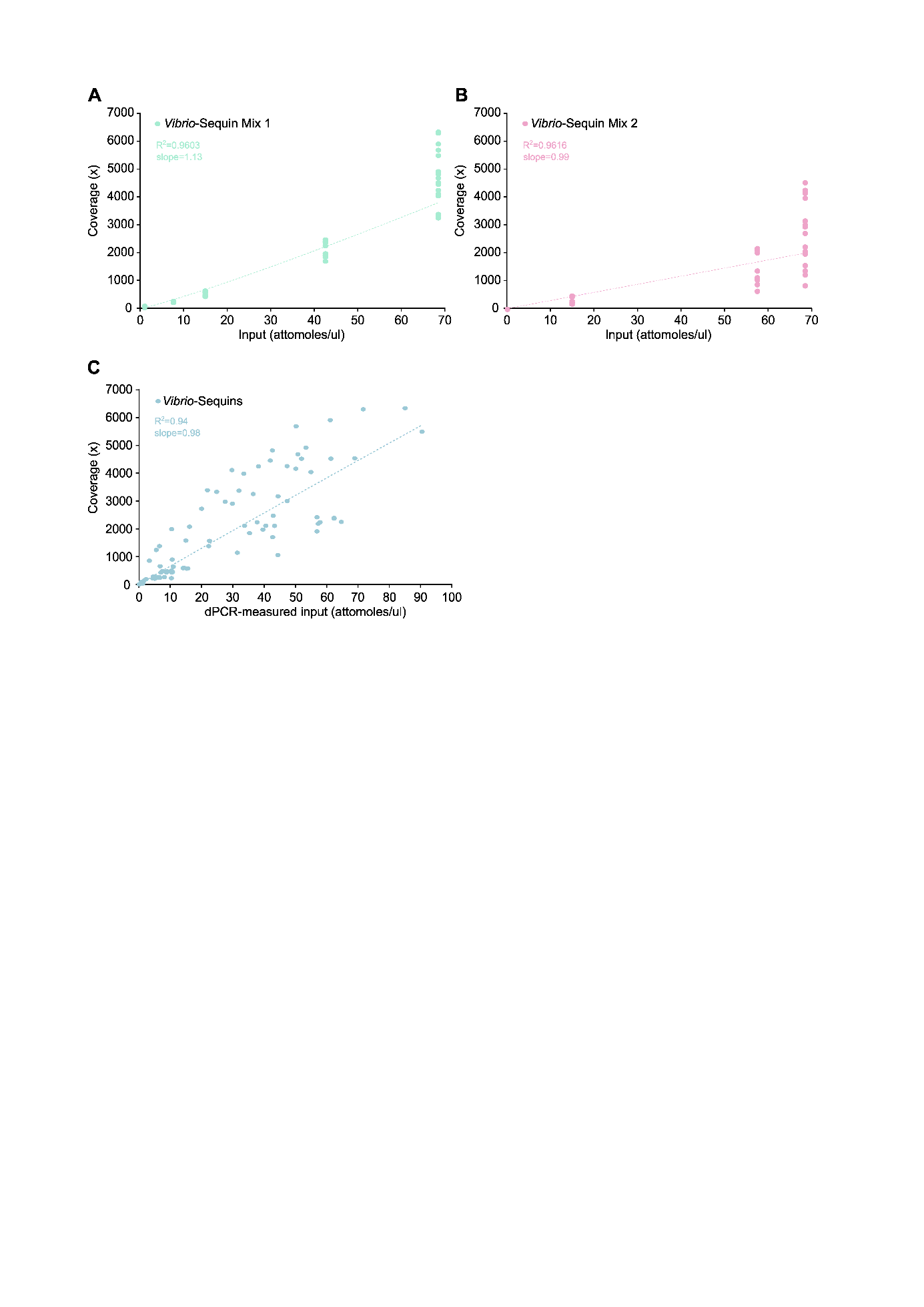
**

**Additional Fig. S6 Coverage of *Vibrio*-sequin mixes 1 and 2 within DNA libraries.** A-B) Quantitative accuracy of *Vibrio*-sequin sequencing within individual DNA libraries (samples S28-S47). Scatter plots showing the unnormalized coverage of individual replicates for each of the six standards HC1, LC1, *rplA*, *ushA*, *valS* and *xni* for both *Vibrio*-sequin mixes A) 1 (samples S28-S37) and B) 2 (samples S38-S47) against the input concentration of the *Vibrio*-sequins (attomoles/µl). C) Scatter plots showing the unnormalized coverage of individual replicates for each of the six standards HC1, LC1, *rplA*, *ushA* , *valS* and *xni* for both *Vibrio*-sequin mixes (1 and 2) within individual DNA libraries (samples S28-S47) against their dPCR-quantified DNA concentrations after library preparation.

**
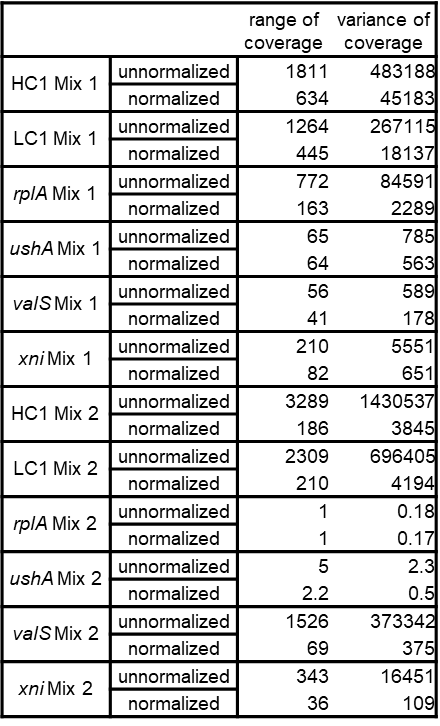
**

**Additional Fig. S7 Variation statistics of coverage normalization.** Shown are the range of datapoints of the coverage along with the variance of coverage before and after normalization using *Vibrio*-Sequins. Data are shown for the individual *Vibrio*-Sequins (HC1, LC1, *rplA*, *ushA*, *valS* and *xni*) in both *Vibrio*-Sequin mixes.
