# Supplementary material for: Vibrio-Sequins - dPCR-traceable DNA standards for quantitative genomics of Vibrio spp
Source: BMC Genomics. 2023 Jul 4;24:375. doi: 10.1186/s12864-023-09429-8 (PMC10318669; doi:10.1186/s12864-023-09429-8)
Supplement: Supplementary file 2 — Additional file 2. Table S1. Information on Vibrio-Sequins. [file 12864_2023_9429_MOESM2_ESM.pdf]

Name	Gene name	Synonym	Gene ID	Description	Length (bp)	GC-content (%)	Reference genome	Chr. Nr.	Sequence similarity Vc & Vp (%)	Sequence similarity Vv & Vp (%)
**HC1**	hypotetical protein	-	partially 1187940	unknown function	540	58	*V. parahaemolyticus* RIMD 2210633 GCA_000196095.1 ASM19609v1	1	-	-
**LC1**	hypotetical protein	-	partially 1187851	unknown function	540	27	*V. parahaemolyticus* RIMD 2210633 GCA_000196095.1 ASM19609v1	1	-	-
rplA	*rplA*	*rpy*	1190500	50S ribosomal subunit protein L1	724	46	*V. parahaemolyticus* RIMD 2210633 GCA_000196095.1 ASM19609v1	1	86	93
ushA	*ushA*	-	1188712	5'-nucleotidase/UDP-sugar hydrolase	1156	44	*V. parahaemolyticus* RIMD 2210633 GCA_000196095.1 ASM19609v1	1	62	64
valS	*valS*	*val-act*	1190191	valine-tRNA ligase	641	46	*V. parahaemolyticus* RIMD 2210633 GCA_000196095.1 ASM19609v1	1	82	91
xni	*xni*	*ygdG, exo, ExoIX*	1188172	flap endonuclease	823	46	*V. parahaemolyticus* RIMD 2210633 GCA_000196095.1 ASM19609v1	1	70	74

Table S1. Information about *Vibrio*-Sequin sequences. Gene ID/name is taken from NCBI, Chr. Nr. = chromosome number, Vc = *V. cholera*, Vp = *V. parahaemolyticus*, Vv = *V. vulnificus.* Names refer to actual gene names (*rplA*, *ushA*, *valS* and *xni*) or to high and low GC content (HC and LC) respectively.
