# Supplementary material for: Vibrio-Sequins - dPCR-traceable DNA standards for quantitative genomics of Vibrio spp
Source: BMC Genomics. 2023 Jul 4;24:375. doi: 10.1186/s12864-023-09429-8 (PMC10318669; doi:10.1186/s12864-023-09429-8)
Supplement: Supplementary file 3 — Additional file 3. Table S2. Oligonucleotides and probes used in this study. [file 12864_2023_9429_MOESM3_ESM.pdf]

Name	Gene ID	Assay type	Fwd primer	Rev primer	Probe	PCR efficiency	Amplicon length	Reference
HC1	partially 1187940	qPCR, dPCR	TCTTCGTGAAAAGCATTCCCA	GAGGCCAAATTTTACAACTATGGTC	Cy5-GCTAACTGCTTCTGTTCAGAACCAATGTT-BHQ2	1.92	194	This study
LC1	partially 1187851	qPCR, dPCR	TCTGTATAGCTGTTGGGAGAACT	CCCCTACCATTGTTATTATCGGT	FAM-AGAAGGGAGTTTTACTTTTTCTAGGCT-BHQ1	1.94	233	This study
rplA	1190500	qPCR, dPCR	ACAGACGCGGTCGAAGAAATCG	GTGCCATGACCGGCGCTGTA	FAM-TGCCATTGGAAGTTCCGCGAAG-BHQ1	1.99	82	This study
ushA	1188712	qPCR, dPCR	TGGTTTACCGACGTGGCAATCATGC	ACTTGAGTCCTCCACAAGCACGTA	Cy5.5-ACCGCCATTCGTTAGCCTGGGCG-BHQ2	1.97	80	This study
valS	1190191	qPCR, dPCR	TGCCATTGGAAGTTCCGCGAAG	TGCCATGTCTGCGTGATGCAA	Cy3-ACCGTCGTTACCGTAGTTGTCCAGCGC-BHQ2	1.86	92	This study
xni	1188172	qPCR, dPCR	TGACCGTCATCGGAAACAGCCA	CGTTCGTACCACCCTCGTTCCGT	Cy5-ACCGCTGGTTTCACCGTTCGGTGCCA-BHQ2	1.99	143	This study
T7 & T3	-	full-insert	TAATACGACTCACTATAGGG	ATTAACCCTCACTAAAGGGA	-	-	-	www.addgene.com/mol-bio-reference/sequencing-primers/

Table S2. Oligonucleotides and probes used in this study. Gene ID/name is taken from NCBI. Names refer to actual gene names (*rplA*, *ushA*, *valS* and *xni*) or to high and low GC content (HC1 and LC1) respectively.
