# Supplementary material for: Vibrio-Sequins - dPCR-traceable DNA standards for quantitative genomics of Vibrio spp
Source: BMC Genomics. 2023 Jul 4;24:375. doi: 10.1186/s12864-023-09429-8 (PMC10318669; doi:10.1186/s12864-023-09429-8)
Supplement: Supplementary file 4 — Additional file 4. Table S3. Sample information. [file 12864_2023_9429_MOESM4_ESM.pdf]

Sample ID	Biosample accession	Description	DNA concentration (ng/ul)	Volume (µl)	Total DNA quantity (ng)	# Reads	Read length (bp)	% GC	% Q30	% Reads remaining after trimming
S1	-	Neat Mix (*rplA, valS, xni*) Rep1	40	30	120	1666082	185	47	100	79
S2	-	Neat Mix (*rplA, valS, xni*) Rep2	40	30	120	1293460	185	47	100	80
S3	-	Neat Mix (*rplA, valS, xni*) Rep3	40	30	120	1810471	185	47	100	80
S25	-	Neat Mix (HC1, LC1,*ushA*) Rep1	40	30	120	1575719	185	33	100	83
S26	-	Neat Mix (HC1, LC1,*ushA*) Rep1	40	30	120	1368394	185	33	100	83
S27	-	Neat Mix (HC1, LC1,*ushA*) Rep1	40	30	120	1902239	185	33	100	83
S28	SAMN32343693	*Vibrio* Mixture A + 2% *Vibrio*-Sequin Mix 1	5.1	30	126	1395356	185	46	100	87
S29	SAMN32343725	*Vibrio* Mixture B + 2% *Vibrio*-Sequin Mix 1	6.78	30	169	1721996	185	46	100	89
S30	SAMN32343726	cultured *V. parahaemolyticus* + 2% *Vibrio*-Sequin Mix 1	4.08	30	122	1718874	185	44	100	88
S31	SAMN32343733	cultured *V. cholerae* + 2% *Vibrio*-Sequin Mix 1	4.08	30	122	1775816	185	47	100	88
S32	SAMN32343735	cultured *V. parahaemolyticus* + 2% *Vibrio*-Sequin Mix 1	4.08	30	122	1460685	185	44	100	89
S33	SAMN32343737	cultured *V. parahaemolyticus* + 2% *Vibrio*-Sequin Mix 1	4.08	30	122	1723683	185	44	100	89
S34	SAMN32343742	Negative *Vibrio* culture + 2% *Vibrio*-Sequin Mix 1	4.08	30	122	1673926	185	44	100	86
S35	SAMN32343743	Negative *Vibrio* culture + 2% *Vibrio*-Sequin Mix 1	4.08	30	122	1863935	185	54	100	90
S36	SAMN32343744	Negative *Vibrio* culture + 2% *Vibrio*-Sequin Mix 1	4.08	30	122	1968015	185	50	100	90
S37	SAMN32343747	Negative *Vibrio* culture + 2% *Vibrio*-Sequin Mix 1	2.75	30	80	1664097	185	38	100	80
S38	SAMN32343748	*Vibrio* Mixture C + 2% *Vibrio*-Sequin Mix 2	2.14	30	54	1045157	185	44	100	88
S39	SAMN32343809	cultured *V. cholerae* + 2% *Vibrio*-Sequin Mix 2	2.75	30	80	580714	185	44	100	88
S40	SAMN32343841	cultured *V. cholerae* + 2% *Vibrio*-Sequin Mix 2	4.08	30	122	1665317	185	47	100	88
S41	SAMN32343897	cultured *V. parahaemolyticus* + 2% *Vibrio*-Sequin Mix 2	4.08	30	122	1522249	185	44	100	91
S42	SAMN32343935	cultured *V. parahaemolyticus* + 2% *Vibrio*-Sequin Mix 2	4.08	30	122	1474037	185	44	100	91
S43	SAMN32343966	cultured *V. cholerae* + 2% *Vibrio*-Sequin Mix 2	4.08	30	122	754171	185	47	100	90
S44	SAMN32344028	Negative *Vibrio* culture + 2% *Vibrio*-Sequin Mix 2	4.08	30	122	643814	185	47	100	90
S45	SAMN32344169	Negative *Vibrio* culture + 2% *Vibrio*-Sequin Mix 2	4.08	30	122	1608306	185	48	100	89
S46	SAMN32344199	Negative *Vibrio* culture + 2% *Vibrio*-Sequin Mix 2	3.75	30	113	1053587	185	41	100	81
S47	SAMN32344254	Negative *Vibrio* culture + 2% *Vibrio*-Sequin Mix 2	4.08	30	122	1248330	185	43	100	88

Table S3. Sequencing sample information. Biosamples are part of the SRA submission PRJNA914529. DNA concentration/quantity concerns sample before library preparation.
