# Supplementary material for: Vibrio-Sequins - dPCR-traceable DNA standards for quantitative genomics of Vibrio spp
Source: BMC Genomics. 2023 Jul 4;24:375. doi: 10.1186/s12864-023-09429-8 (PMC10318669; doi:10.1186/s12864-023-09429-8)
Supplement: Supplementary file 8 — Additional file 8. Information on amplicon location. [file 12864_2023_9429_MOESM8_ESM.docx]

**Amplicon location information for the TaqMan Assays of the *Vibrio*-Sequins**

> *V. parahaemolyticus*_GCA_000196095.1_ASM19609v1 BA000031.2:479500-480000, **HC1,** 540 bp - inverted

5'̵TAATACGACTCACTATAGGGTTCGACCTATAAATATAGAACAAAATAAAAAAATTATTGACATAAAAATTTATTTTCTTTCAATAGTGTGTTTTTTCTTCGTGAAAAGCATTCCCAATATTAAAGGAAAAGTTAGTATTGTTAACATCGCGGTTGACGTGAATACAAAGAATGATTTCTTGTGAATTATTCTGTTCCAATAAGAAAGAGCTAACTGCTTCTGTTCAGAACCAATGTTCTATCTCAAATATAAAAATTTTTCATTGACCATAGTTGTAAAATTTGGCCTCATAAACCATGTAATTTTGCATGAAAAGAAAAACCTTCCATATTTTGAATCAGTGTGTATTCTTTTGAATTTTATTGCGTCTTTTAGGTAATTTTATTAATTGTATAGTTCTCATATAAAAATGTAAATTGTGACTCTGGTATGGACTTTTATTTTATTATTAAATTTTATTTATTCGGGTAAAGTAAGTTACTTATCTTAAGTAACGTATAGAAAGATAATACTAAGCATTTCCCTTTAGTGAGGGTTAAT-3'

FWD Primer

REV Primer

Probe

Flanking regions for full-length amplification (T7 and T3 primer sites)

> *V. parahaemolyticus*_GCA_000196095.1_ASM19609v1 BA000031.2:385000-385500, **LC1,** 540 bp – inverted

5'̵TAATACGACTCACTATAGGGAGAAAAGAAATTATAGAAAAGAAAAGTTTGCTTGAAAACCTTGTAACAAGAAAAGTAGATTTAATTATCTTAAACGAGACGCATTACGAAGAAGTAGACTTTAAAGTAACATCTAGTGAAATTTTAATAACTCGAATCAAAGTGCAAGAAGAGAATGCCTATCTATTGAGTAAAGTGCTAATGCAAATCTTATTTGGTCTTGAAATCTGTATAGCTGTTGGGAGAACTATATATCTATTTAAGTTACTGGTAATATAAATAGTTCATTATAGGAGAAACATTTCCTGGAAAATTATGATAATTTTTAGTGAAATTGTAAATTAATAATTTATATTTAGCAAACAAAAAAATTAATAATTTAATCTATTTATCGTTAATTAAAGTGAGAAGGGAGTTTTACTTTTTCTAGGCTAATACCGATAATAACAATGGTAGGGGGATTGATTTTTATTAGTATAAGTAGTAGTATTAAAGACTCAATCCTAGGATTGGCTCTTACTTCCCTTTAGTGAGGGTTAAT-3'

FWD Primer

REV Primer

Probe

Flanking regions for full-length amplification (T7 and T3 primer sites)

>NC_004603.1:c1268879-1267185 *V. parahaemolyticus* RIMD 2210633 chromosome 1, ***ushA,*** 1156 bp - inverted

5'̵TAATACGACTCACTATAGGGGATAAGTCGACGCTAAAGTGTTGGTTCACCCGACAGACTCAATCACATCTGTTAAAGCGGAACTCCAAGCATTAACGAATGAACATACATAGCTCTCGCAGATCTATCGACATGTAATGAAGACGTAGTTGCAATGGTCACAATTAATGCGATTCGCGACCCATTGGTAGTGGCGGTCGACGCAACTTCGAACCATGCCATTTAAACATTCAACGACAACGCAGCTTTAGAAACGGCAACTAGAATGACTAAAGTTGAACGAATCGACATGTCAGATGCCAGTATAAATCTGGACGATCAACACGTATGCGTGGACTCGGTGCAACGTAGCATCGCTGATGCAAGTTCATTAGCTACTGAAGTCGTGGTCAGTACCATCGATGACAATGTCATAATGGTTTACCGACGTGGCAATCATGCACCGCCATTCGTTAGCCTGGGCGAAGTTAACTACGTGCTTGTGGAGGACTCAAGTAATGTCGTTTTAGACGAAAAACTAAAAGGACTACTCGTCAACGCTAGTCTGCTGGATTCAACCAAACAACTATCGCTTGGTGTCGTGCCAGTGGAAGGTTAAATGGTAGTCTTAAACGTTAATGGAATAGATCGAGTGGAACAGGAAACGAAACTTTTATAAGTTCTGCAAGAACTTCTAGTTCCAGACCAAACTAAAGTCATGGTTACTTGAGATCGAGTGGACGAAGCAAAAATGCAAAATTCAAATGACCTTGATCGAGTATTGGTCTTTCCACTTCAAATGGCAGTATCATAAGCTTCAGTCGAAATGGTTGCATGAATGGGGTAAGCACACGAACGTATTACATTCATGGCAAAACAACCAGACCAAATGTCAGTAGTGGACCGAGCTTCAGGCGTATAGGACTTGGTAGTCCAAGGTATGTTTGACCCAAAACGCTCACTGGTGGTTTCTATCGACGTTCAACAGGAAGAAGCTGTCGAAATCGGTAACGATGTAGTGGTCCCCGTAAACTTGGCACGACTGGTAAAAGTATCACTGGGTATACGCAGCATCGCTTTTATTTTAGCTGTCCCAGTAGAAACATCCAAAGAAACTAAAGACGTTATTGGAAGCCGTAAAGAAGGCATCCGAACCATTCCCTTTAGTGAGGGTTAAT-3'

FWD Primer

REV Primer

Probe

Flanking regions for full-length amplification (T7 and T3 primer sites)

>NC_004603.1:c3119085-3118384 *V. parahaemolyticus* RIMD 2210633 chromosome 1, ***rplA,*** 724 bp - inverted

5'̵TAATACGACTCACTATAGGGAATACGAACTCACAAGTCCGAGCGGACTAGTTGTCGTTGTGGTCGTGGGTATCATCATCTCTACGAATGAAAGAAGTCCTTTCATGGAAAGCGACTTCTACCGAATCGAAAGAAGTCTCGTTGATCTTCACGAAGATCCAAGAGAAACTAGACGAGTCGTCTCTTCAAACGAAATGGCTATCATCACACCTACTACGGCAAAAACAGCAATGCCATTGCTTGGACTGGTCGAAATCGCAAAAATTGACGAAGTCGTTGCAATCCTCAATGTCATGGTTGAAATCCCAAACCGTATTCTGGCGCTCCTGGATCCTATCATGGATCAACTGGATGTTGCGCGTAACGTAGACCTCTTCGTTGTTGATGCAGTTTCAAGTAAAGCGGAAAGAATTGAACGAGTCGTTCTAGAAGGTATGGTTGCTACAGACGCGGTCGAAGAAATCGACGAAGTCGCAAACGTGGAACTCACTTGTGACGATGCGCCTACAGCGCCGGTCATGGCACTCCATCATGTCAACGTGGTGCATGCAAGACCAGTCTAAATGCTCGTAGTTACGGATCCAATTGTCGTTGCAGATGTCTGAGTTGCTTAAATCGTCATCGATCAAGAACTTCTTCTCGTTGTCGAAGCAACTAAAGCATAAGAAATCAATGCAGTTGAAAAAGCGCCTAATGCGCGTACGCTCCCTTTAGTGAGGGTTAAT-3'

FWD Primer

REV Primer

Probe

Flanking regions for full-length amplification (T7 and T3 primer sites)

>NC_004603.1:2798087-2800945 *V. parahaemolyticus* RIMD 2210633 chromosome 1, ***valS***, 641 bp - inverted

5'̵TAATACGACTCACTATAGGGAATCGAGATCCATGGTCAGTGTCTTAACCAAGGTCTACTTAAGCATGTCGCACAATCTGCGGTATAGATCTGCCTTCAACAGCTATACCAACAACTTGCGAAAGCGACGTTCAAGTTTGACTCTGAGCTAGGTGAACAGGCGATCACTCATAAGCTAGAGTGGTGGCAACTTCGGTGTCAGAACGAGAAGACACAAGTAGTAATGCATTGCCGATCGCAAGGTATCAAACAATGTCTTCAATGCCATTGGAAGTTCCGCGAAGTATAGGGTCAACTATAGCGCTGGACAACTACGGTAACGACGGTCGCACTTTGCATCACGCAGACATGGCATACGAAGCTATGGCAAAAGTTTGCAGAATGCTCATAAGAAGAGCTAGAAGAAGCGATCGACTCCAACGTAGTACAACGGTCACGCGAAGAGATGATCTCTGAGATCCAGCTATGGTAGCTAGTACAGCTAACCCAGTTCGTGCAATGGAAAGCTGAATCTGTAGAACAGCGGCAAAAGCAGTGCTTAATCCGGCCATTGCATTTGGCAAAATTTACCATGAACACCGAACGGCAAAAGCAGAAAGTGCTTCACGTACCAGTAGTACTCCCTTTAGTGAGGGTTAAT-3'

FWD Primer

REV Primer

Probe

Flanking regions for full-length amplification (T7 and T3 primer sites)

>NC_004603.1:c3119085-3118384 *V. parahaemolyticus* RIMD 2210633 chromosome 1, ***xni***, 823 bp - inverted

5'̵TAATACGACTCACTATAGGGAATGAGCCGAAATAAACCAGGCCATTTCGCTTACAGAACGTCCAATTTCGGCTCGAGCTATAGCGTAAAGTTGCGGCGATGAGCAAATGTATCTGCACGTCTGAGTTACACAAGTAGGTCGAAGAACGCTATAAAGCCACGTTCAAGTAGCGACCGCATCCGACGAAGTTATAGAAGTTTAACTCAATCCTAGAGAAACCGCCGAAAGCCAGGTTACGGGCCTTGCGGACCGTGAACCGAACTTGATTACGGTCAATTGGGGGTTATTAGTCGCTCAACCGAACCGAAATGCGGTTTGAGAAAGAGTTATTTGCCAAGTAGGTTGGTTGCCACAACTTTCATTAGCGCTTAAACGTCACAGCCACTTTCATTGACCGTCATCGGAAACAGCCAACTTTGCTAGCAATGAAAGAGTGGCACCGAACGGTGAAACCAGCGGTCACAACGTTGATTTAGCAGGCGGAGTAGTGGAAGACTATCGTCACTTAGTTACGGAACGAGGGTGGTACGAACGACCTACCGTAGGTTTGGAACGTAGTCGCCGAGACCGTAGCCAAACGCTAAAACGAACATCAGTCCTTCCTAAAGTCGCGCGGTTGGAGATAGAACAAGTACCACTAGTTTGTGGCGCTACTATACACAACCAAAGCGAAGCCTTTCTTATGCGCATTCCCAAGCGACTCAGCAGCACTACCAAGAACGCTACAGTCAACCTAGGCCAACACGTCTCACGTGTGCCGCTTAGTTCAACTCCCGTAGTTATTATTGTTCTACTTATCTGTATCCCTTTAGTGAGGGTTAAT-3'
